# Efficacy and safety of adipose tissue-derived cell therapy in the treatment of chronic extremity ulcers: a systematic review and meta-analysis of randomized controlled trials

**DOI:** 10.1590/1677-5449.202501392

**Published:** 2026-09-14

**Authors:** Edenilson de Souza Teixeira, Vanessa Ellen Silva Carmo, Hideki Zimermann Kamitani, Johnnatas Mikael Lopes, Sydney Correia Leão, Carlos Alberto de Lima Botelho, Pedro Pereira Tenório

**Affiliations:** 1 Universidade Federal do Vale do São Francisco, Paulo Afonso, BA, Brazil.; 2 Universidade Federal do Rio Grande do Norte, Natal, RN, Brazil.; 3 Universidade de Ciências da Saúde de Porto Alegre, Porto Alegre, RS, Brazil.; 4 Universidade Federal de Pernambuco, Recife, PE, Brazil.

**Keywords:** mesenchymal stem cells, wound healing, cell and tissue-based therapy, chronic pain

## Abstract

**Context:**

Chronic extremity ulcers represent a major clinical challenge. Adipose-derived stem cells (ADSCs) have emerged as a promising therapeutic option for wound healing; however, their efficacy and safety remain incompletely established.

**Objectives:**

To evaluate the efficacy and safety of ADSCs compared with conventional treatment.

**Methods:**

A systematic review and meta-analysis of 11 randomized clinical trials involving 474 patients was conducted. Outcomes included healing rate, mean healing time, pain-related outcomes, and safety. Odds ratios (ORs) and weighted mean differences were used as summary effect measures.

**Results:**

Complete healing rates were significantly higher in the ADSC group than in the conventional treatment group (84.2% vs. 49.0%; OR 5.77; p < 0.00001). Healing time was reduced by 19.39 days on average in ADSC group (p = 0.0004). No statistically significant differences were observed regarding pain outcomes or complication rates (p = 0.22**).**

**Conclusion:**

ADSC therapy increased healing rates and shortened healing time in patients with chronic extremity ulcers, with no evidence of a significant increase in treatment-related risks. However, the heterogeneity of the findings and the limited sample size highlight the need for studies with greater statistical power and methodological standardization to confirm these results.

## INTRODUCTION

Chronic ulcers are defined as lesions that did not progress appropriately through the wound-healing process, failing to regain anatomical and functional integrity within the expected timeframe of up to 3 months.^1,2^ These lesions are commonly associated with conditions such as diabetes mellitus (DM), venous insufficiency, pressure injuries, and arterial diseases, and are characterized by high prevalence (2.21 cases per 1,000 inhabitants), substantial treatment costs, and a significant impact on quality of life.^3-6^ Impaired healing results from factors such as tissue ischemia, persistent infection, and dysregulated inflammatory responses, which impair normal tissue repair mechanisms, leading to increased rates of amputation and mortality.^4,5^ In this context, adipose-derived stem cells (ADSC) therapy has emerged as a promising approach to stimulate tissue regeneration and promote the healing of chronic ulcers.^7^

ADSCs are multipotent mesenchymal stem cells capable of differentiating into various cell types, such as fibroblasts, endothelial cells, and keratinocytes. Furthermore, they secrete growth factors, cytokines, and anti-inflammatory mediators that regulate the wound microenvironment.^8^ These properties make ADSCs particularly attractive for the treatment of chronic ulcers, in which restoration of vascularization, reduction of inflammation, and promotion of extracellular matrix deposition are essential for wound healing. Recent clinical studies have shown that ADSC-based therapies can accelerate wound closure, enhance granulation tissue formation, and reduce the risk of recurrence.^9^

Adipose tissue is an abundant and readily accessible source of mesenchymal stem cells, facilitating their clinical application. ADSC therapy can be administered through local injections, topical application, or incorporation into biomaterials such as hydrogels and scaffolds, which facilitate controlled release and retention of cells within the wound bed.^10^ In this context, a systematic review and meta-analysis was conducted to evaluate the available evidence regarding the use of ADSCs in chronic extremity ulcers and their potential benefits compared with conventional treatment (debridement, wound cleansing, and dressings), with a focus on healing rate, healing time, pain modulation, and safety profile. Understanding these aspects is essential for establishing the role of ADSCs as an effective and safe therapeutic tool in the management of complex and hard-to-heal wounds.

## METHODS

This systematic review and meta-analysis were conducted in accordance with the Preferred Reporting Items for Systematic Reviews and Meta-Analysis (PRISMA) guidelines.^11,12^ and the Cochrane Collaboration Risk of Bias 2 (RoB 2) tool.^13^ The review protocol was prospectively registered with the International Prospective Register of Systematic Reviews (PROSPERO) under registration number CRD420251002247.

### Eligibility criteria and data extraction

Only studies meeting the following criteria were included: (1) randomized controlled trials investigating the impact of ADSCs; (2) studies using autografts or allografts that were considered eligible for analysis; (3) studies involving patients with hard-to-heal chronic extremity ulcers; and (4) assessment of at least one of the following outcomes: healing rate, healing time, amputation rate, mortality, infections, quality of life, and pain. Non-randomized studies, animal studies, reviews, letters to the editor, and opinion articles without original data were excluded, as were trials involving patients treated with adipose tissue-derived cells for acute wounds or wounds not affecting the extremities, and patients with terminal illnesses or contraindications to regenerative therapy. No restrictions were imposed regarding study population size.

Data were extracted according to predefined search criteria and quality assessment methods, with studies reviewed at two separate stages to minimize the risk of inappropriate exclusion. Data extraction and validation were performed independently by two reviewers, and any discrepancies were resolved by consensus among three authors. Information collected included patient characteristics, ulcer characteristics, type of intervention, healing rate, healing time, and procedure-related outcomes. These data subsequently underwent additional review and pooled analysis. The primary outcomes were healing rate and healing time, whereas secondary outcomes included pain modulation and the safety profile associated with ADSC therapy.

### Search strategy

A systematic search was conducted in PubMed, EMBASE, Cochrane Central Register of Controlled Trials, and Scopus, from database inception through December 2024 and was limited to studies published in English. The following search terms were used: ("Complex wounds" OR "Chronic wounds" OR "Non-healing wounds" OR "Pressure ulcers" OR "Diabetic foot ulcers" OR chronic OR ulcer OR diabetic) AND ("Adipose tissue therapy" OR "Adipose-derived stem cells" OR adipose OR "Fat grafting" OR "Cell therapy" OR "Stem cell therapy" OR "Regenerative therapy" OR "Regenerative medicine" OR "lipoaspirate cells") AND ("Amputation" OR "Mortality" OR "Infection" OR healing OR "Tissue repair" OR "Tissue regeneration" OR "Wound closure" OR "Quality of life"). The reference lists of the included studies, as well as relevant meta-analyses and review articles, were manually screened to identify additional potentially eligible studies not captured by the initial search strategy.

### Quality assessment

Risk of bias and methodological quality were assessed using the RoB 2 tool^13^ for randomized controlled trials. Each study was classified as having a low risk, some concerns, or high risk of bias across five specific domains: the randomization process, deviation from intended interventions, missing outcome data, outcome measurement, and selection of the reported results. Moreover, funnel plots were constructed to assess the potential presence of publication bias by examining the relationship between study estimates and their corresponding measures of precision.

### Statistica analysis

Odds ratios (ORs) and corresponding 95% confidence intervals (95%CIs) were calculated for all binary outcomes. Statistical heterogeneity was assessed using Cochran’s Q test and Higgins I^2^ statistics, whereas publication bias was evaluated using Egger’s test and funnel plots. Low heterogeneity was defined as p > 0.10 and I^2^ < 25%. Pooled estimates were calculated using the DerSimonian-Laird random-effects model, with p < 0.05 considered as statistically significant. A random-effects model was applied even in the absence of significant statistical heterogeneity because of the clinical heterogeneity among studies. This approach assumes that pela true effects are not identical across studies and that, if it were possible to perform an infinite number of studies, the effect estimates of all studies would follow a normal distribution.^14^. Statistical analyses were performed using Review Manager 5.4 (Nordic Cochrane Centre, The Cochrane Collaboration, Copenhagen, Denmark).^15^

### Assessment of certainty of the evidence

The certainty of the evidence was assessed using the Grading of Recommendations Assessment, Development and Evaluation (GRADE) approach.^16^ The assessment considered five domains: study limitations (risk of bias), inconsistency of results, indirectness of evidence, imprecision, and publication bias. The certainty of evidence for each outcome was categorized as high, moderate, low, or very low. The findings were presented in a summary of findings table.

## RESULTS

As shown in Figure 1, the database search strategy, supplemented by manual screening of reference lists from relevant reviews and meta-analyses, initially identified 1,474 records. After removal of duplicates and exclusion of records deemed irrelevant based on title and abstract screening, 30 articles were selected for full-text review and assessed according to the predefined inclusion and exclusion criteria. Of these, 19 studies were excluded for the following reasons: eight were non-randomized, five consisted of conference abstracts from studies already included or failed to meet the eligibility criteria, five had study designs without published results, and one was a duplicate. Ultimately, 11 studies involving a total of 474 patients were included in the analysis. Of these, 247 patients (52.1%) received ADSC treatment for chronic ulcers, whereas the remaining 227 patients (47.9%) received standard treatment consisting of debridement, wound cleansing, and dressings.^17-27^

**Figure 1:**
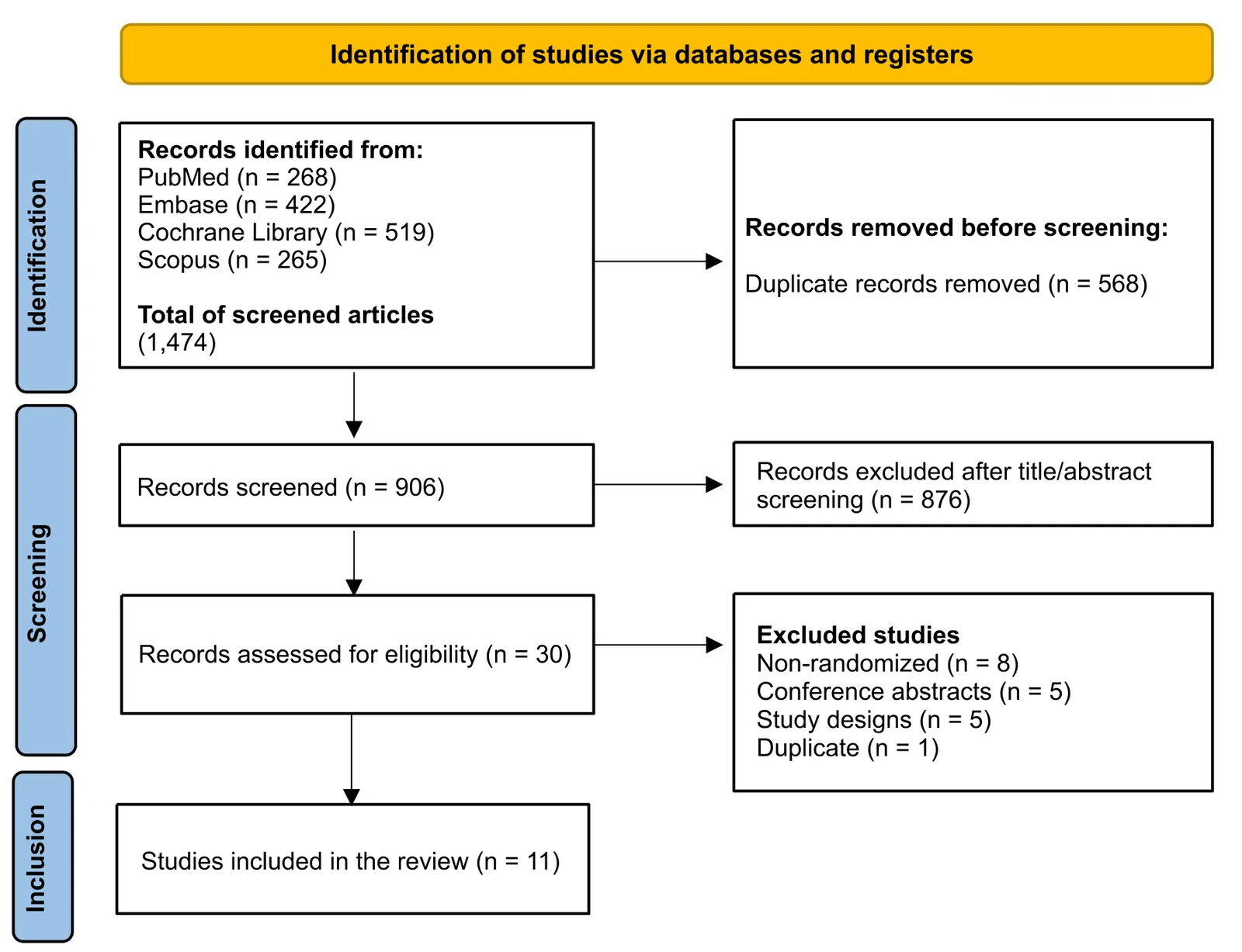
Preferred Reporting Items for Systematic Reviews and Meta-Analyses (PRISMA) flow diagram of study screening and selection.

Assessment of methodological quality using the RoB 2 tool indicated that four controlled clinical trials were judged to be at high risk of bias, as shown in Figure 2. In the randomization process domain, the study by Zollino et al.^17^ raised some concerns, whereas that by Alinda et al.^26^ presented a high risk of bias in the randomization process, both because information on allocation concealment was not reported. In the study by Zollino et al.,^17^ a high risk of bias in outcome measurement was also identified due to lack of information regarding blinding of outcome assessors, limiting the assessment of the adequacy of randomization procedures. Namgoong et al.^18^ presents a high risk of bias in outcome measurement due to non-blinded assessment and the use of subjective clinical judgments. Added to this is the failure in allocation concealment (Domain 1), which compromises randomization, and the lack of blinding of participants and personnel (Domain 2), which raises additional concerns, although the intention-to-treat analysis was adequate. The lack of pre-registration of the protocol and statistical analysis plan also raises doubts regarding transparency and outcome selection (Domains 4 and 5). Taken together, these factors substantially compromise confidence in the study's effect estimates. Similarly, the study by Uzun et al.^22^ showed high risk of bias in the selection of reported results by reporting only statistically significant results among multiple subscales without adjustment for multiple comparisons, in addition to high risk of bias in the randomization process, resulting from uncertainties in allocation concealment. These methodological limitations may affect the internal validity of the respective studies. Nevertheless, their overall impact was mitigated by the predominance of trials judged to be at low risk of bias.

**Figure 2:**
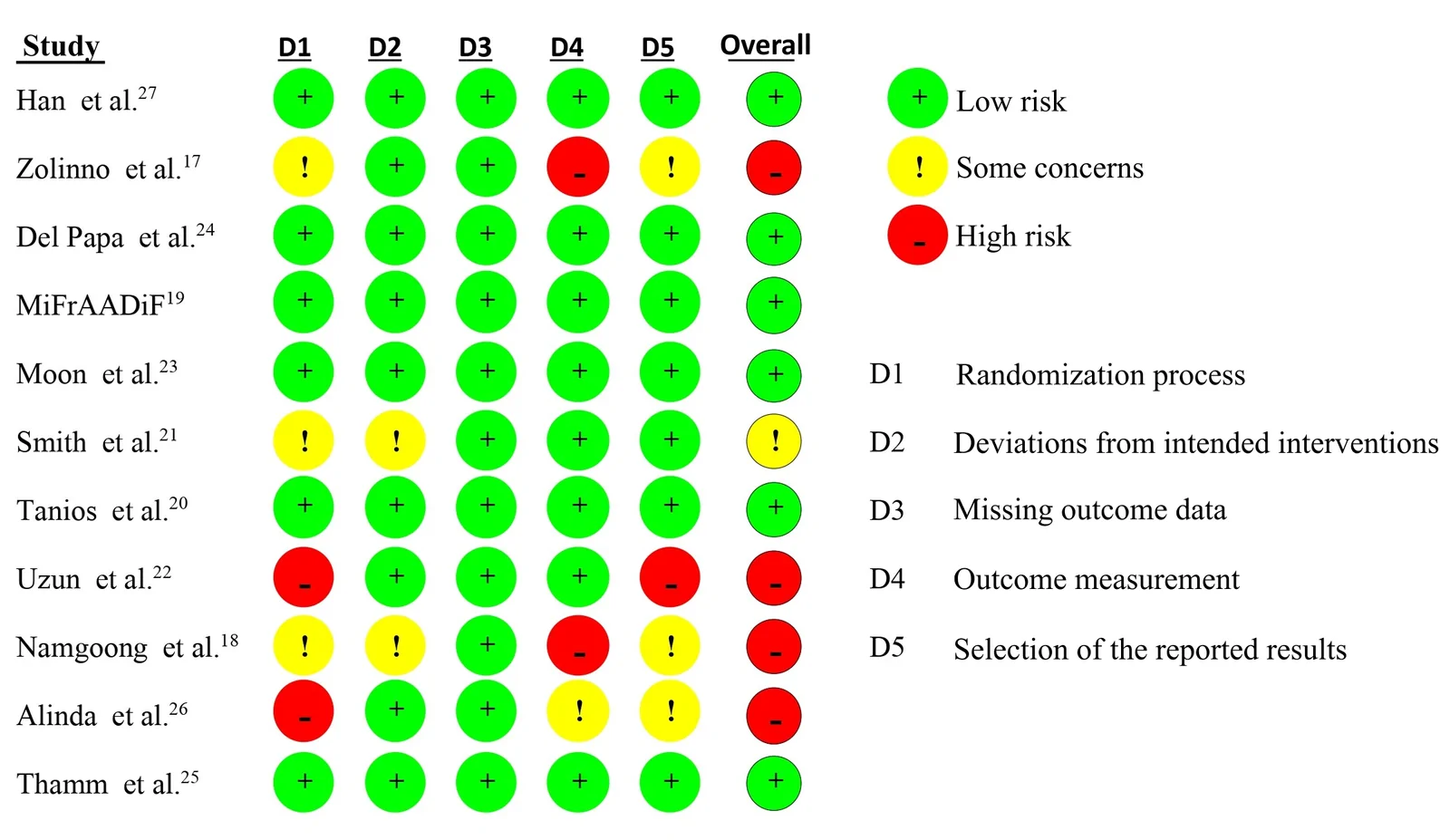
Critical assessment of individual studies according to the Cochrane Risk of Bias 2 (RoB 2) tool for randomized controlled trials.

Patient characteristics are detailed in Table 1. The included studies showed heterogeneity regarding the route of ADSC administration: two clinical trials used topical application,^23,26^ whereas nine employed local injection.^17-22,24,25,27^. Moreover, the cell source varied across studies, with eight using autologous adipose tissue,^17-22,24,25,27^ two using allogenic adipose tissue,^22,23^ and one not specifying the source of the cells.^26^ The most common adipose tissue harvest sites were the lower and lateral abdomen, as well as the medial and lateral tight. Follow-up duration ranged from 2 to 50 months. Regarding ulcer etiology, approximately 71% of cases were associated with DM. Other causes included leprosy, systemic sclerosis, venous ulcers, trophic ulcers, arterial ulcers, and other causes.

**Table 1:** Characteristics of the included studies.

**Study**	**Patients**	**Age (years)**		**Sex (M/F)**		**Ulcer etiology**			**Wound size (cm^2^)**		**Duration of ulcer (weeks)**	**Follow-up duration (weeks)**	**ABI**
		ADSC	Control	ADSC	Control	ADSC	Control		ADSC	Control			
**Han et al. (2010)** **^27^**	52	66.5±7.5	68.4±8.7	15/11	14/12	DM			4.3±2.1	4.0±2.1	12.5±5.5	8	> 0.5
**Zollino et al. (2018)** **^17^**	16	74±6.7	68±12.8	5/3	5/3	75% VU/25% mixed	62.5% VU/37.5% mixed		31.3±59.0	29.0±42.0	≥ 36	24	> 0.85: VU; < 0.85: mixed
**Del Papa et al. (2019)** **^24^** ^*^	38	42 (21-69)	37 (23-70)	2/23	0/13	Systemic sclerosis			0.75 (0.65–0.82)^†^	0.73 (0.62–0.84) †	ADSC: 11.4 (9.7-13.1) Control: 8.0 (7.1-8.8)	8	N/A
**MiFrAADiF (2019)** **^19^**	114	69.0±11.6	71.6±10.8	45/12	41/16	DM			N/A	N/A	N/A	24	≥ 0.7
**Moon et al. (2019)** **^23^**	59	59.9±13.3	68.4±9.9	14/8	13/4	DM			2.0±0.9	2.8±2.0	35.6±75.2	12	0.7 < 1.3
**Smith et al. (2020)** **^21^** ^*^	12	60.2 (45-78)	55.2 (41-69)	6/0	4/2	DM			3.4 (1.1-4.9)	5 (1.2-17.5)	49 (8-132)	12	≥ 0.3
**Tanios, et al. (2021)** **^20^**	100	48.12±14.58	48.04±10.56	29/21	24/26	70% DM, 24% TU, 2% VU, 4% post-trauma		84% DM, 16% TU	6.28±0.98	5.63±0.75	47.6±33.21	24	N/A
**Uzun et al. (2020)** **^22^**	20	57.5±8.4	57.2±4.5	6/4	6/4	DM			23.5.6	25.8±5.4	7.4±2.7	208.6‡	55% (< 0.8); 45% (≥ 0.8)
**Namgoong et al. (2022)** **^18^**	20	59.3±9.92	56.9±11.39	8/2	9/1	DM			5.66±4.74	4.66±6.86	≥ 6	16	N/A
**Alinda et al. (2023)** **^26^**	32	45.75±4.97	45.19±7.22	10/6	9/7	Leprosy			1.67±1.52		> 6	8	N/A
**Thamm et al. (2023)** **^25^**	31	62±11	60±14	13/4	13/1	35.3% VU, 11.7% AU, 17.6% DM, 0% PU, 47% other	14.2% VU, 21.4% AU, 14.2% DM, 14.2% PU, 42.8% other		6.3±6.2	4.8±4	105±130	8.57	N/A

ABI = ankle-brachial index; AU = arterial ulcer; DM = diabetes mellitus; M/F = male/female; N/A = not applicable; PU = pressure ulcer; TU = trophic ulcer; VU = venous ulcer. Data are presented as mean and standard deviation.

* Age, wound size, and ulcer duration are presented as mean (interquartile range).

† Wound size presented as median (interquartile range), in centimeters.

‡ Mean follow-up duration: 208.6 weeks (interval: 113.0–217.3 weeks).

### Contribution of ADSCs on wound healing rates

Ten studies evaluated the effectiveness of ADSC-based therapy on wound healing rates compared with conventional treatment, with follow-up periods ranging from 6 to 24 months. Complete wound healing was observed in 84.2% of patients receiving ADSC therapy, compared with 49.0% of those in the control group.

Subgroup analysis was performed for DM-related ulcers and cutaneous ulcers of mixed etiologies. Among the six clinical trials evaluating DM-related ulcers, the pooled consistently favored ADSC therapy and reached a high level of statistical significance (OR 4.56; 95%CI 2.40-8.67; p < 0.00001), with no observed heterogeneity (I^2^ = 0%). In the subgroup of ulcer with mixed etiologies, ADSC therapy was likewise associated significantly greater likelihood of healing than standard treatment (OR 9.04; 95%CI 2.60-31.36; p = 0.0005), although moderate-to-high heterogeneity was observed across studies (I^2^ = 52%).

The pooled analysis of clinical trials confirmed the overall benefit of ADSC therapy (OR 5.77; 95%CI 3.34-9.96; p < 0.00001), with low between-study heterogeneity (I^2^ = 9%). No statistically significant differences were identified between subgroups (p = 0.34), indicating that the therapeutic benefit was maintained across both DM-related ulcers and ulcers of diverse etiologies. Taken together, these findings suggest that ADSC therapy is a promising intervention for the healing of cutaneous ulcers, with particularly robust and homogeneous evidence in the setting of diabetic ulcers, as shown in Figure 3. Egger’s test did not indicate the presence of publication bias, yielding a bias coefficient of 0.593 (95%CI -1.363 to 2.550; p = 0.504). This finding was further supported by visual inspection of the funnel plot shown in Figure 4.

**Figure 3:**
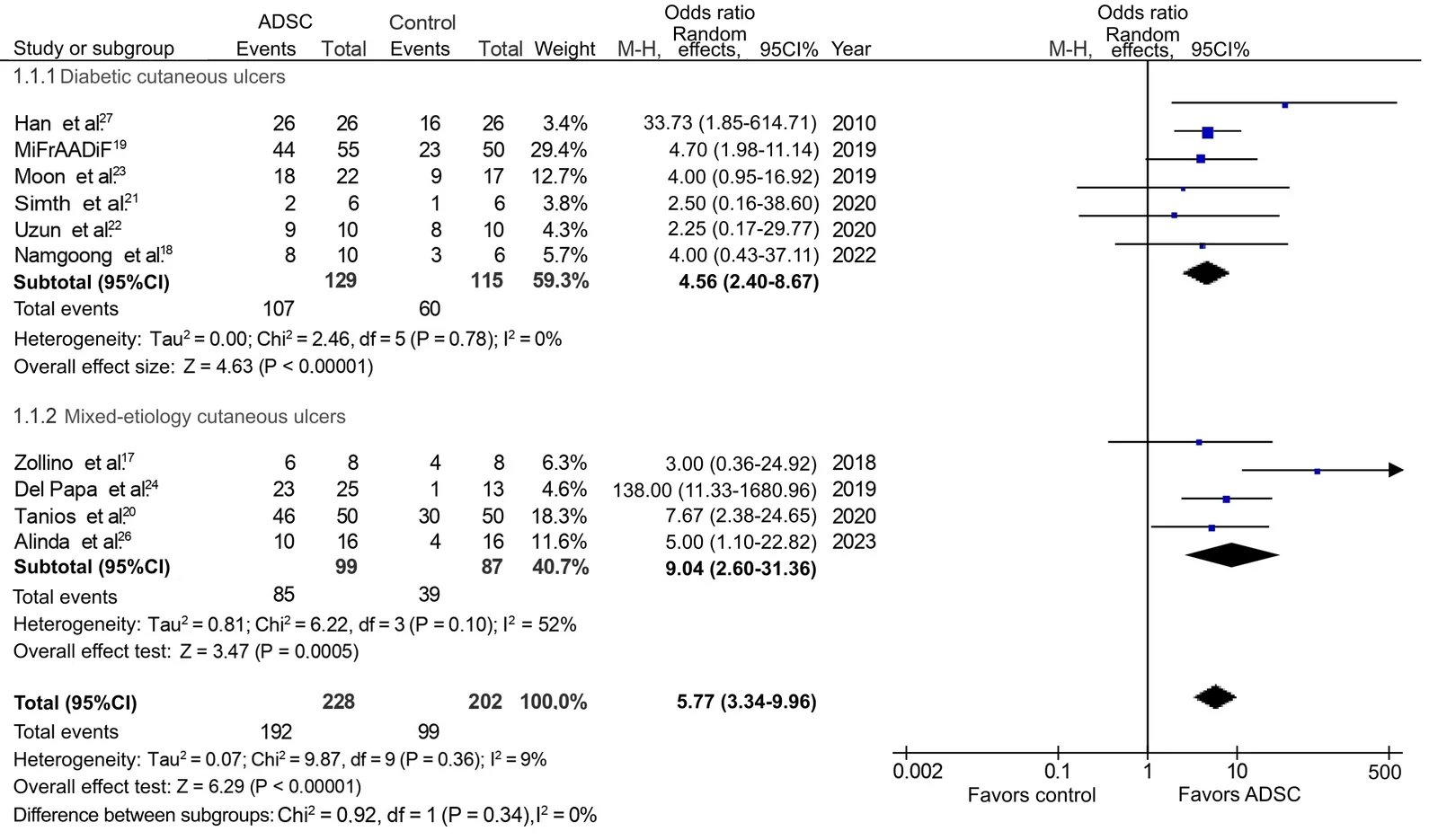
Forest graph showing significantly higher ulcer healing rates in the adipose-derived stem cell therapy group. CI = confidence interval; df = degrees of freedom; DM = diabetes mellitus; M-H = Mantel-Haenszel.

**Figure 4:**
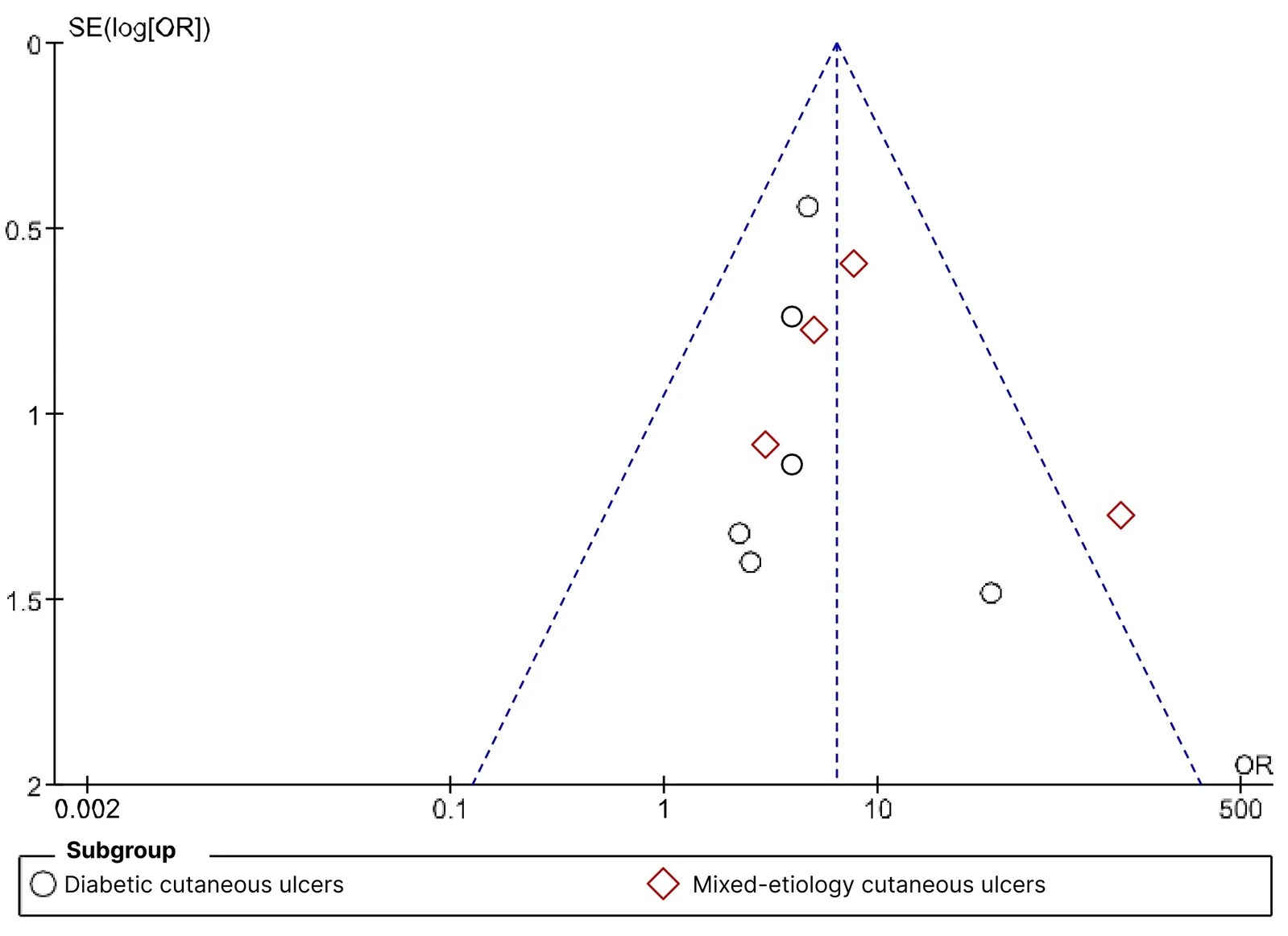
Funnel plot for the assessment of publication bias in healing rates. DM = diabetes mellitus.

Subgroup analysis according to cell source among trials involving patients with DM revealed that the autologous ADSC subgroup (four studies; 190 participants) demonstrated a significant treatment effect (OR 5.69; 95%CI 2.72-11.90; p < 0.00001; I^2^ = 0%). In the allogenic ADSC subgroup (two studies; 59 participants), a trend toward benefit was observed, with borderline statistical significance (OR 3.49; 95%CI 0.99-12.29; p = 0.05; I^2^ = 0%). No statistically significant differences were detected between subgroups (p = 0.51; I^2^ = 0%), suggesting that the therapeutic effect of ADSCs did not differ significantly according to cell source.

After exclusion of trials classified as having a high risk of bias according to the ROB-2 tool, the sensitivity analysis demonstrated that both statistical significance and the overall effect remained unchanged, yielding a pooled OR of 7.79 (95%CI 3.25-18.67; p < 0.00001; Z = 4.60). Between-study heterogeneity remained low (I^2^ = 23.3%; p = 0.25), indicating consistent findings. In the subgroup analysis by ulcer etiology, the subgroup of mixed-etiology cutaneous ulcers (two studies; 138 participants per group) showed an OR of 26.11 (95%CI 1.59-429.31), indicating a favorable treatment effect. In the diabetic ulcer subgroup (four studies; 208 participants), the pooled effect estimated was OR = 4.86 (95%CI 2.43-9.74; p < 0.0001), with no evidence of heterogeneity (I^2^ = 0%; p = 0.55), further supporting the robustness of the evidence in this population.

Analysis of the individual ORs demonstrated statistically significant treatment effects favoring ADSC therapy in five studies.^19,20,24,26,27^ Notably, the study by Del Papa et al. ^24^ reported an OR of 138.00 (95%CI 11.33-1,680.96), indicating a string treatment effect, despite the extremely wide confidence interval. Similarly, Han et al.^27^ found an OR of 33.73 (95%CI 1.85-614.71), suggesting a favorable effect of the intervention. Furthermore, the MiFrAADiFL^19^ study and that by Tanios et al.^20^ reported statistically significant ORs of 4.70 (95%CI 1.98-11.44) and 7.67 (95%CI 2.38-24.65), respectively. In contrast, Moon et al.^23^ (OR 4.00; 95%CI 0.95-16.92) and Smith et al.^21^ (OR 2.50; 95%CI 0.16-38.60) yielded imprecise estimates, as reflected by their wide confidence intervals, likely because of their small sample sizes.

### Impact of ADSCs on healing time

Seven studies provided sufficient data for the analysis of healing time. The mean healing time ranged from 31 to 122 days among patients treated with ADCSs (n = 183) and from 39 to 171 days among control participants (n = 178). Pooled analysis showed that ADCS therapy significantly shortened healing time by 19.39 days in the intervention group (95%CI -30.17 to -8.61; p = 0.0004), as illustrated in Figure 5.

**Figure 5:**
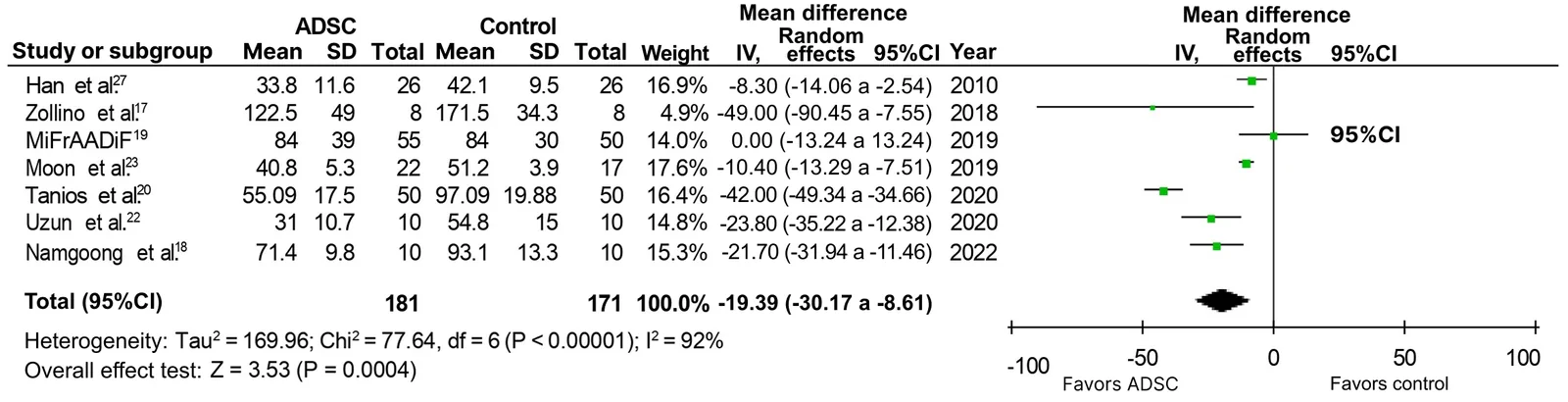
Forest graph showing significantly lower mean healing time in the adipose-derived stem cell therapy group. CI = confidence interval; df = degrees of freedom; IV = inverse variation; M-H = Mantel-Haenszel; SD = standard variation.

Substantial heterogeneity was observed in the analysis of healing time, with Tau^2^ = 169.96. Cochran’s Q statistics was 77.64 (degrees of freedom [df] = 6; p < 0.00001), and Higgins I^2^ was 92% (95%CI 81.4%-98.4%), confirming substantial statistical heterogeneity and considerable inconsistency across studies. The funnel plot (Figure 6) showed a wide dispersion of effect estimates, consistent with the high degree of heterogeneity observed.

**Figure 6:**
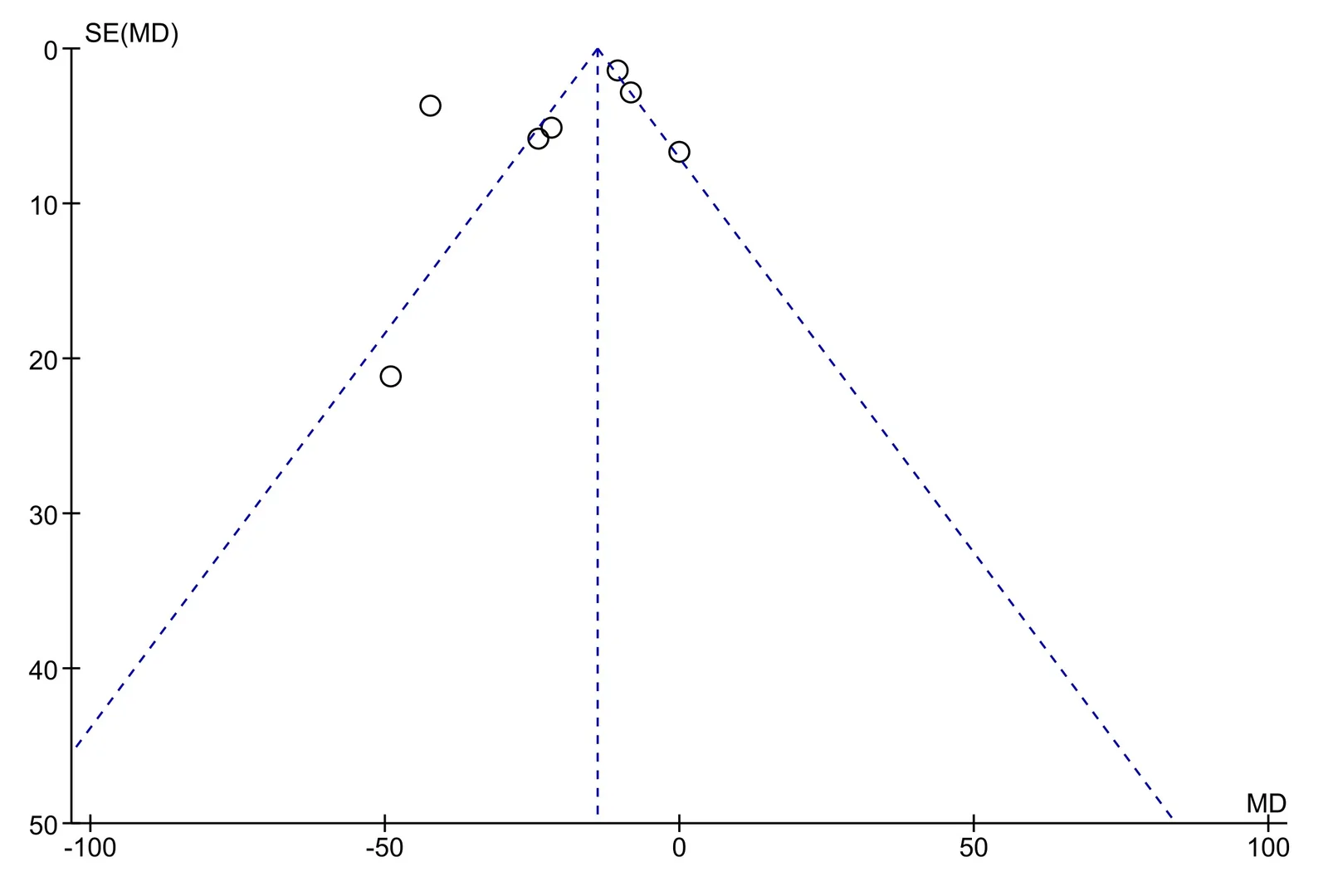
Funnel plot for the assessment of publication bias in mean healing time.

Subgroup analysis limited to diabetic ulcers (five studies; 236 participants) demonstrated that ADSC was associated with a mean difference of -12.44 days in healing time (95%CI -18.33 to -6.55; p < 0.0001), despite substantial between-group heterogeneity (I^2^ = 68%; p = 0.01). When stratified according to cell source, the autologous ADSC subgroup (four studies; 197 participants) showed a mean difference of -13.52 days (95%CI -23.39 to -3.64; p = 0.007), also with substantial heterogeneity (I^2^ = 76%; p = 0.006). Sensitivity analysis excluding studies at high risk of bias confirmed the robustness of treatment effect, with a mean difference of -15.55 days (95%CI -30.11 to -0.98; p = 0.04), although heterogeneity remained high (I^2^ = 96%; p < 0.00001). Overall, these findings indicate considerable variability across studies, which may be attributable to differences in cell administration protocols and follow-up duration.

Individually, the study by Zollino et al.^17^ reported the largest mean difference in healing time (-49.00 days; 95%CI -90.45 to -7.55), despite the broad confidence interval. Significant reductions in healing time were also reported by Tanios et al.^20^ (mean difference: -42.00 days; 95%CI -49.34 to -34.66) and Namgoong et al.^18^ (mean difference: -21.70 days; 95%C -31.94 to -11.46). Conversely, the MiFrAADiF^19^ study found no significant difference between the intervention and control groups (mean difference: 0.00 days; 95%CI -13.24 to 13.24).

### Contribution of ADSC therapy in pain modulation

Five studies evaluated the effects of ADSC therapy on pain using the Visual Analog Scale (VAS), the Numeric Rating Scale (NRS), or the pain domain of the 36-item Short Form Health Survey (SF-36).^17,19,22,24,25^ Of these, two studies demonstrated statistically significant results favoring ADSC therapy.^17,24^

Del Papa et al.^24^ observed a reduction of more than 50% in pain intensity in 21 of 25 patients treated with adipose tissue extract, whereas none of the 13 patients in the control group experienced a comparable improvement after 8 weeks (p < 0.00001, Fisher’s exact test). Likewise, Zollino et al.^17^ reported that the NRS score decreased to 2.7±2.0 during the first week after treatment with autologous adipose-derived cells, compared with 6.6±3.0 in the control group (p < 0.01).

Conversely, the MiFrAADiF^19^ study found no significant differences between groups at any follow-up visit. A significant reduction in pain was observed over time (p < 0.001); however, treatment with microfragmented adipose tissue also contributed significantly to this outcome (p < 0.05). Similarly, Thamm et al.^25^ and Uzun et al.^22^ reported modest reductions in pain in both groups over time, although these changes did not reach statistical significance.

### Safety profile related to ADSC therapy

Of the included clinical trials, nine provided sufficient data for the assessment of procedure-related adverse events. Two studies reported adverse events in the ADSC-treated group,^17,19^ whereas the remaining studies identified no complications associated with the intervention.^18,20,22-24,26,27^ Overall, three adverse events were reported among 222 patients receiving ADSC therapy, compared with no events among 200 patients in the control group.^18,20,22-24,26,27^.

In one cases, perilesional dermatitis was observed short after cell administration and resolved spontaneously by the second week of follow-up.^17^ In the MiFrAADiF study, two abdominal wall hematomas at the adipose tissue harvest site were reported: one was successfully managed with a compression dressing, whereas the other required surgical incision to achieve hemostasis of the subcutaneous tissue. Both patients were receiving oral anticoagulant therapy.

In a three-arm study conducted by Smith et al.,^21^ one patient developed a superficial fluid collection along the graft cannula tract, requiring drainage. However, the study did not specify to which treatment arm the patient had been assigned, whether the adipose tissue treatment group (the intervention of interest in the present review), or to the third arm, which received fat grafting combined with platelet-rich plasma).

The analysis yielded an OR of 4.07 (95%CI 0.42-38.91), suggesting a numerically higher risk of complications in the ADSC group; however, the wide confidence interval indicates substantial uncertainty around the estimate. The overall effect test (Z = 1.23; p = 0.22) did not demonstrate a statistically significant difference between groups. Furthermore, no statistically significant heterogeneity was observed in the safety analysis across the studies (Q = 0.02; df = 1; p = 0.89; I^2^ = 0%), as shown in Figure 7.

**Figure 7:**
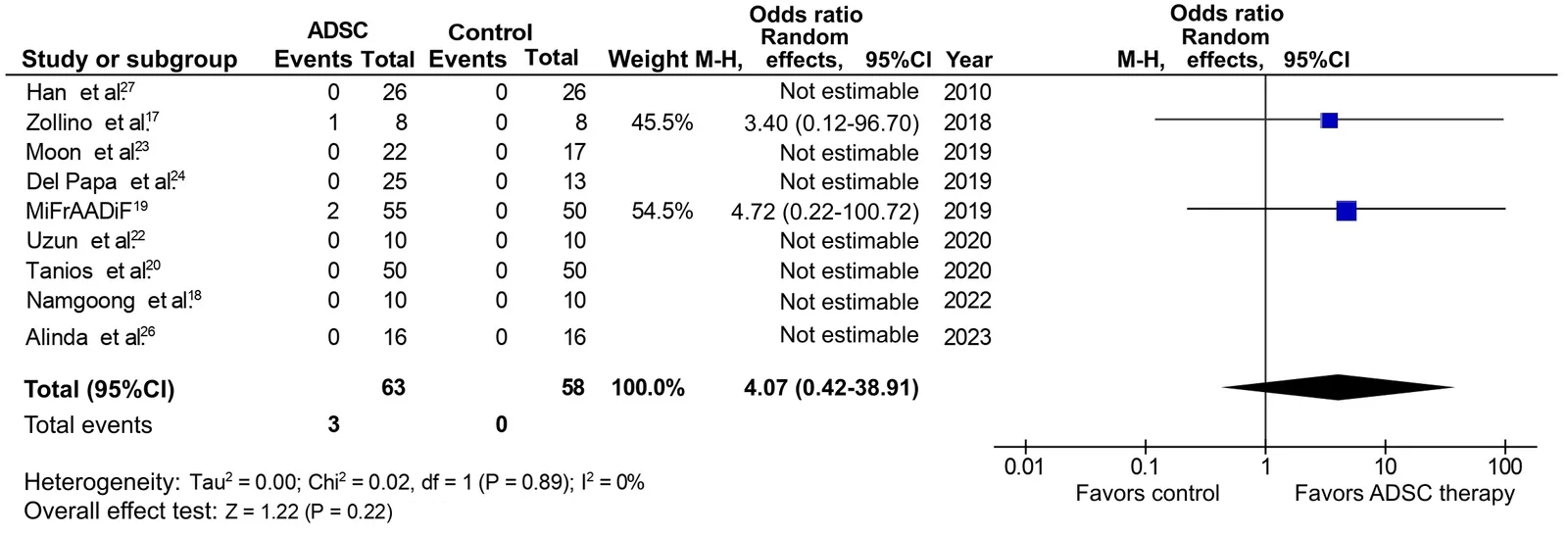
Adipose-derived stem cell therapy demonstrated a favorable safety profile, with no significant increase in the risk of complications compared with standard therapy. CI = confidence interval; df = degrees of freedom; M-H = Mantel-Haenszel.

Therefore, although adverse events were reported among patients who received ADSC therapy, the current evidence does not demonstrate a statistically significant increase in the risk of complications compared with standard therapy.

### Assessment of certainty of evidence

Certainty of evidence was assessed using the GRADE approach for the outcomes of healing rate, healing time, and safety (Table 2). For healing rate, the primary analysis yielded moderate-certainty evidence, downgraded for indirectness because of clinical heterogeneity in ulcer etiology and in intervention protocols. In the analysis restricted to patients with DM, the certainty of evidence remained moderate but was downgraded for imprecision due to the low number of events (< 300). Regarding healing time, certainty was rated as very low certainty for both the analysis including ulcers of mixed etiologies and the DM-specific analysis owing to substantial heterogeneity (I^2^ > 50%) that was not explained by subgroup analyses. The certainty of evidence for safety was also rated as very low, owing to very low inconsistency (overlapping confidence intervals), indirectness, and very serious imprecision (wide confidence intervals crossing the line of no effect).

**Table 2:** Assessment of the certainty of evidence using the Grading of Recommendations Assessment, Development and Evaluation (GRADE) approach.

**Certainty of evidence**							**No. of patients**		**Effect**		**Certainty**	**Importance**
**No. of studies**	**Study design**	**Risk of bias**	**Inconsistency**	**Indirectness**	**Imprecision**	**Other considerations**	**ADSC**	**Control**	**Relative (95%CI)**	**Absolute (95%CI)**		
**Ulcer healing rate without etiological stratification**												
10	Randomized clinical trials	Not serious	Not serious	Serious^*^	Not serious	None	192/228 (84.2%)	99/202 (49.0%)	OR 5.77 (3.34-9.96)	357 more events per 1,000 patients (272 more to 415 more)	⊕⊕⊕◯Moderate^*^	Critical
**Ulcer healing rate in patients with DM**												
6	Randomized clinical trials	Not serious	Not serious	Not serious	Serious^†^	None	107/129 (82.9%)	60/115 (52.2%)	OR 4.56 (2.40-8.67)	311 more events per 1,000 patients (202 more to 383 more)	⊕⊕⊕◯Moderate^†^	Critical
**Ulcer healing time without etiological stratification**												
7	Randomized clinical trials	Not serious	Very serious^‡^	Serious^*^	Not serious	None	181	171	-	MD: 19.39 fewer days (30.17 fewer to 8.61 fewer)	⊕◯◯◯Very low*^‡^	Important
**Ulcer healing time in patients with DM**												
5	Randomized clinical trials	Not serious	Very serious^‡^	Not serious	Serious^†^	None	123	113	-	MD: 12.44 fewer days (18.33 fewer to 6.55 fewer)	⊕◯◯◯Very low†‡	Important
**Complications related to ADSC therapy**												
9	Randomized clinical trials	Not serious	Very serious^§^	Serious^*^	Very serious	None	3/63 (4.8%)	0/58 (0.0%)	OR 4.07 (0.42-8.91)	0 per 1,000 patients (0 to 0)	⊕◯◯◯Very low*§||	Important

CI = confidence interval; MD = mean difference; OR = odds ratio.

* Population including patients with ulcers of different etiologies (diabetic, venous, arterial, autoimmune, and leprosy-related) and treated using different ADSC administration methods (topical application vs. injection; autologous vs. allogeneic cells).

† Fewer than 300 events.

‡ Substantial heterogeneity (I^2^ > 50%).

§ Overlapping confidence intervals across studies.

|| The pooled-effect diamond crosses the line of no effect and is associated with a wide confidence interval.

## DISCUSSION

Mesenchymal stem cells (MSCs) have emerged as a promising cell-based therapeutic approach for the treatment of chronic wounds.^28,29^ These cells can be obtained from various sources, including bone marrow, adipose tissue, and umbilical chord tissue. ADSCs represent a population of MSCs isolated from adipose tissue. Numerous *in vitro* and *in vivo* studies have demonstrated the benefits of MSCs, which include the secretion of angiogenic cytokines, promotion of epithelization and granulation tissue formation, and addition anti-inflammatory and antiapoptotic effects, all of which contribute to improved ulcer healing.^19,30^

ADSCs and related therapies, such as cell sheets, autologous and allogeneic cell, and exosomes, may significantly improve wound healing by promoting tissue regeneration, neovascularization, and granulation tissue formation, while reducing inflammation and improving skin quality in diabetic, venous, trophic, and pressure ulcers.^30-33^

The present meta-analysis showed that complete healing was achieved in 83.3% of patients treated with ADSCs, compared with 48.5% of those receiving standard care consisting of debridement, wound cleansing, and dressings, indicating a statistically significant difference. Similar findings were reported by Elsharkawi et al.^5^ in a meta-analysis evaluating ulcer healing in patients with DM. In that study, which included 189 patients followed for up to 12 months, complete healing was achieved in 83.5% of patients treated with ADSCs, compared with 52% of those in the control group. A favorable effect of the intervention has also been observed in ulcers of other etiologies. Del Papa et al.^24^ found that 92% of patients with systemic sclerosis who received adipose tissue grafts achieved healing of digital ulcers after 8 weeks, compared with only 7.7% of patients in the control group.

ADSC therapy has shown promising results in the treatment of wounds in patients with DM.^34^ Han et al.^27^ reported complete wound healing in all patients treated with processed human lipoaspirate-derived cells, compared with 62% of patients in the control group. Moon et al.^23^ used allogeneic ADSCs derived from healthy donors and incorporated into hydrogel cell sheets for the treatment of diabetic foot ulcers, achieving complete wound closure in 82% of treated patients compared with 53% of controls after 12 weeks (p = 0.053). Collectively, these findings suggest that ADSCs, particularly when delivered as cell sheets, may enhance wound healing by promoting vascularization, reducing fibrosis, and restoring skin architecture.^35^

Although statistical heterogeneity for healing rate was low (I^2^ = 9%), the clinical heterogeneity among studies should be carefully considered, particularly with regard to ulcer etiology. The pathophysiology of wound healing differs substantially among venous, arterial, diabetic, leprosy-related, and autoimmune ulcers, reflecting distinct underlying mechanisms. As a result, combining these diverse etiologies into a single analysis, even when statistical heterogeneity is low, may mask clinically relevant differences in treatment effect. The overall benefit associated with ADSC therapy may therefore not be consistent across all ulcer subtypes, and the magnitude of the effect may vary according to the underlying etiology. Therefore, caution is warranted when generalizing these findings across different wound populations, especially given the limited statistical power of subgroup analysis resulting from reduced sample sizes.

Furthermore, the magnitude of the observed effect (OR 5.77) should be interpreted with caution. Studies with wider confidence intervals and smaller sample sizes tended to report the most extreme estimates, a pattern that is consistent with potential small-study effects.^36^ Although such findings may be associated with methodological limitations and the preferential publication of positive results, the available evidence does not allow this possibility to be confirmed. Consequently, some degree of overestimation of the treatment benefit cannot be ruled out.

Patients receiving ADSC therapy experienced a shorter healing time, with a pooled standardized mean difference (SMD) of -19.35 (95%CI -30.12 to -8.58). The study by Tanios et al.^20^ reported the largest effect estimate, with an SMD of -42.03 (95%CI -49.37 to -34.69). In that study, the mean healing time was 7.87±2.50 weeks in the ADSC group compared with 13.87±2.84 weeks in the control group (p = 0.000), with injections administered every 3 weeks.

Interventions such as those reported by Tanios et al.^20^ and Namgoong et al.^18^ demonstrated the potential to accelerate wound healing and provide clinically meaningful benefits. In the analysis restricted to patients with diabetic ulcers, ADSC therapy showed a consistent benefit, with an approximate 12-day reduction in healing time. This finding is clinically important, given that diabetic foot ulcers are among the leading causes of morbidity, hospitalization, and nontraumatic amputations.^37^ Shortening healing time may improve patients’ quality of life while reducing healthcare costs.

The substantial heterogeneity observed in the meta-analysis of patients with DM for the outcome of healing time (I^2^ = 68%) likely reflect multiple sources of variability, including differences in ulcer severity, adjunctive treatment protocols (debridement, antibiotic therapy, and pressure offloading), sample size, and follow-up duration. Studies reporting shorter healing times in the control group may have included less severe ulcers, whereas those involving patients with a poorer baseline prognosis appeared to derive greater absolute benefit from the intervention. The MiFrAADiF^19^ study, the only trial showing no treatment benefit, may reflect specific methodological or population-related characteristics. Nevertheless, its inclusion did not alter the direction of the overall effect and primarily contributed to increased between-group variability.

Despite the observed variability, the directional consistency of the findings – with all studies except one favoring the intervention – supports a beneficial effect of ADSC therapy on reducing healing time in diabetic ulcers. The treatment effect appeared to be more pronounced in analyses involving autologous cells; however, the substantial heterogeneity observed in both the overall and subgroup analysis limits the precision of the pooled estimate. Moreover, the limited number of studies precluded meta-regression analyses to explore specific sources of inconsistency, such as ulcer size, presence of infection, or glycemic control.

A relevant secondary outcome reported by Zollino et al.^17^ and Del Papa et al.^24^ was a significant reduction in pain, reflected by immediate improvements in NRS and VAS scores, respectively, following cellular therapy. Chronic venous ulcers are associated with impaired quality of life, largely because of persistent and difficult-to-control pain. Stem cell therapy may modulate the local cytokine cascade within the wound environment, exerting anti-inflammatory effects, as suggested by the secretome hypothesis and the immunomodulatory microenvironment established by stem cells.^31^

Conversely, the MiFrAADiF^19^ study found no significant differences between groups with respect to pain reduction, indicating that improvements were primarily driven by a significant time effect (p < 0.001). However, pain improvement appeared to occur earlier in the treatment arm. The authors suggested that the absence of between-group differences may be explained by the high prevalence of distal diabetic polyneuropathy, which complicates the differentiation between neuropathic pain and pain directly attributable to the wound.^19,38^ Supporting the potential analgesic properties of adipose-derived therapies, studies osteoarthritis have demonstrated significant reductions in VAS scores following intra-articular administration of microfragmented adipose tissue.^39^

With respect to safety, ADSC administration by injection, cell-sheet delivery, or topical application demonstrated a favorable safety profile. In the MiFrAADiF srudy,^19^ seven deaths were reported during follow-up in addition to the previously described abdominal wall hematomas; however, none of these deaths were considered treatment related. Several clinical trials also reported lower infection rates among treated ulcers. Tanios et al.^20^ found that only 6% of patients in the treatment group developed infection, compared with 28% in the control group (p = 0.000). This effect may related to the biological activity of stem cells and the enhanced vascularization associated with angiogenesis.^19,30^

In the clinical trial reported by Han et al.,^27^ two participants were excluded because of clinically evident infections that occurred 5 and 6 weeks after treatment. Moon et al.^23^ reported serious adverse events, including cellulitis on untargeted sites, paresthesia, uncontrolled DM, and cardiac arrest. Similarly, Uzun et al.^22^ observed complications in 15% of participants, such as recurrent infections, necrosis, and minor amputations. None of these events was considered related to ADSC therapy or to the wound dressings used in the studies.

The cost-effectiveness of ADSC therapy for chronic extremity ulcers should be assessed not only on the basis of the initial product cost but also in terms of the total episode-of-care cost. Health economic studies have demonstrated that cellular and tissue-based therapies can represent cost-effective treatment options when evaluated over an appropriate time horizon. Carter and Fife^40^ proposed objective for interpretation, whereby interventions costing ≤ US$ 140 per cm^2^ are considered economically dominant (providing superior outcomes at lower overall cost) relative to standard care, whereas costs > US$ 430 per cm^2^ exceed the willingness-to-pay threshold of US$ 100,000 per quality-adjusted life year (QALY) gained and are therefore unlikely to be economically justified. Furthermore, Nherera & Banerjee^41^ showed, in a 1-year economic model, that all evaluated cellular products were cost-effective relative to standard care, with total costs ranging from US$ 10,907 to US$ 19,498 compared with US$ 19,862 for conventional treatment. These findings suggest that the initial investment may be offset by reductions in complications such as infections and amputations.

Within the specific context of ADSC therapy, the findings reported by Uzun et al.^22^ and Smith et al.^21^ are consisted with this perspective. Although the upfront costs of treatment may be higher, potential benefits such as earlier return to work, a lower incidence of osteomyelitis, and reduced amputation rates may translate into overall healthcare savings. However, the variability of costs among similar products highlights the need for incorporating economic evaluations into clinical trials to determine whether ADSCs therapies fall within established cost-effectiveness thresholds (US$ 140-US$ 430/cm^2^) and to identify the patient subgroups (such as those with larger or refractory ulcers) in whom the clinical benefits justify the investment.

In light of the evidence analyzed in this study, and considering the limitations identified, ADSC therapy was associated with favorable therapeutic outcomes in the treatment of chronic extremity ulcers. According to the GRADE approach, the certainty of evidence was moderate for the healing outcome and was more robust among patients with diabetic ulcers treated with autologous cells. Evidence for healing rate was classified as low because of the limitations inherent to this review and the limited number of available randomized clinical trials. Studies considered at high risk of bias did not substantially influence the meta-analysis results. Important limitations of the present study include the small sample size and the pooling of chronic extremity wounds without detailed etiological stratification, which may have introduced residual clinical heterogeneity.

## CONCLUSION

The use of ADSCs in the treatment of chronic extremity ulcers appears promising, with the potential to significantly reduce healing time and increase healing rates, particularly in diabetic ulcers and when autologous cell-based approaches are used. The available evidence does not indicate a significant increase in risk associated with ADSC harvesting or administration procedures. However, the methodological heterogeneity across studies warrants caution when interpreting the findings and applying them to broader populations. Although four clinical trials were classified as having high risk of bias according to the RoB 2 tool, their outcomes were consistent with those reported in studies at low risk of bias, reinforcing the overall favorable direction of the evidence. Nevertheless, additional studies with larger sample sizes, randomized designs, greater methodological rigor, and standardized protocols are needed to confirm these findings and further define the role of ADSCs in the management of chronic wounds.

## DISPONIBILIDADE DE DADOS

Todos os dados gerados ou analisados estão incluídos neste artigo e/ou no material suplementar.
