# Supplementary material for: Efficacy and safety of adipose tissue-derived cell therapy in the treatment of chronic extremity ulcers: a systematic review and meta-analysis of randomized controlled trials
Source: J Vasc Bras. 2026 Sep 14;25:e20250139. doi: 10.1590/1677-5449.202501392 (PMC13671007; doi:10.1590/1677-5449.202501392)
Supplement: Translation

# Translation: Eficácia e segurança da terapia com células derivadas do tecido adiposo no tratamento de úlceras crônicas de extremidades: uma revisão sistemática e metanálise de ensaios clínicos randomizados e controlados

**DOI:** 10.1590/1677-5449.202501391

**Authors:** Edenilson de Souza Teixeira, Vanessa Ellen Silva Carmo, Hideki Zimermann Kamitani, Johnnatas Mikael Lopes, Sydney Correia Leão, Carlos Alberto de Lima Botelho, Pedro Pereira Tenório

**Affiliations:** 1 Universidade Federal do Vale do São Francisco, Paulo Afonso, BA, Brasil.; 2 Universidade Federal do Rio Grande do Norte, Natal, RN, Brasil.; 3 Universidade de Ciências da Saúde de Porto Alegre, Porto Alegre, RS, Brasil.; 4 Universidade Federal de Pernambuco, Recife, PE, Brasil.

**Keywords:** células-tronco mesenquimais, cicatrização de feridas, terapia baseada em transplante de células e tecidos, dor crônica

## Abstract

**Contexto:**

As úlceras crônicas de extremidades representam um importante problema de saúde na prática clínica. As células-tronco derivadas do tecido adiposo (ADSCs) surgem como uma alternativa promissora para promover a cicatrização dessas lesões, embora sua eficácia e segurança ainda não estejam definitivamente estabelecidas.

**Objetivos:**

Avaliar a eficácia e a segurança das ADSCs em comparação com o tratamento convencional.

**Métodos:**

Foi realizada revisão sistemática e metanálise de 11 ensaios clínicos randomizados, envolvendo 474 pacientes. Foram avaliados a taxa e o tempo de cicatrização, os desfechos relacionados à dor e a segurança. As medidas de efeito incluíram *odds ratio* (ORs) e diferença média ponderada.

**Resultados:**

A taxa de cicatrização completa foi maior no grupo tratado com ADSCs (84,2% vs. 49,0%; OR 5,77; p < 0,00001). O tempo de cicatrização foi, em média, 19,39 dias menor no grupo intervenção (p = 0,0004). Não houve diferença estatisticamente significativa em relação à dor ou à incidência de complicações (p = 0,22).

**Conclusão:**

As ADSCs aumentaram a taxa de cicatrização e reduziram o tempo necessário para a cicatrização de úlceras crônicas de extremidades, sem evidência de aumento significativo do risco associados ao tratamento. Contudo, a heterogeneidade dos achados e o tamanho amostral limitado reforçam a necessidade de estudos com maior poder estatístico e protocolos metodológicos padronizados para confirmar esses resultados.

## INTRODUÇÃO

As úlceras crônicas são definidas como lesões que não evoluíram adequadamente ao longo do processo de reparo tecidual, falhando em restaurar a integridade anatômica e funcional dentro do período esperado de até 3 meses^1,2^. Estão frequentemente associadas a condições como diabetes melito (DM), insuficiência venosa, lesão por pressão e doenças arteriais, apresentando alta prevalência (2,21 casos por 1.000 habitantes), elevado custo terapêutico e impacto significativo na qualidade de vida^3-6^. Sua cicatrização inadequada resulta de fatores como isquemia tecidual, infecção persistente e resposta inflamatória desregulada, que comprometem os processos naturais de reparo tecidual, aumentando as taxas de amputação e mortalidade^4,5^. Nesse contexto, a terapia com células-tronco derivadas do tecido adiposo (*adipose-derived stem cells*, ADSCs) tem surgido como uma abordagem promissora para estimular a regeneração tecidual e promover a cicatrização de úlceras crônicas^7^.

As ADSCs são células-tronco mesenquimais multipotentes com capacidade de se diferenciar em diversos tipos celulares, incluindo fibroblastos, células endoteliais e queratinócitos. Além disso, secretam fatores de crescimento, citocinas e moléculas anti-inflamatórias capazes de modular o microambiente da ferida^8^. Essas propriedades tornam as ADSCs particularmente atrativas para o tratamento de úlceras crônicas, nas quais a restauração da vascularização, a redução da inflamação e o estímulo à deposição de matriz extracelular são fundamentais para o reparo tecidual. Estudos clínicos recentes demonstram que a administração de ADSCs pode acelerar o fechamento da lesão, favorecer a formação de tecido de granulação e reduzir o risco de recorrência^9^.

O tecido adiposo é uma fonte abundante e de fácil acesso para a obtenção de células-tronco mesenquimais, facilitando sua aplicação clínica. A terapia com ADSCs pode ser realizada por meio de injeções locais, aplicação tópica ou incorporação em biomateriais, como hidrogéis e *scaffolds*, que auxiliam na retenção celular e em sua liberação controlada no leito da ferida^10^. Nesse contexto, realizou-se uma revisão sistemática e metanálise com o objetivo de analisar as evidências disponíveis sobre a aplicação de ADSCs em úlceras crônicas de extremidades e seus potenciais benefícios em comparação com o tratamento convencional (desbridamento, limpeza de ferida e curativos), com foco na taxa e no tempo de cicatrização, na modulação da dor e na avaliação do perfil de segurança do procedimento. A compreensão desses aspectos é fundamental para consolidar o papel das ADSCs como uma ferramenta terapêutica eficaz e segura no manejo de feridas complexas e de difícil cicatrização.

## MÉTODOS

Esta revisão sistemática e a metanálise foram conduzidas em conformidade com as recomendações do Preferred Reporting Items for Systematic Reviews and Meta-Analysis (PRISMA)^11,12^ e com as diretrizes da ferramenta de avaliação de risco de viés Risk of Bias 2 (RoB 2) da Cochrane Collaboration^13^. O protocolo da revisão foi registrado prospectivamente no International Prospective Register of Systematic Reviews (PROSPERO) sob o número de protocolo CRD420251002247.

### Critérios de elegibilidade e extração dos dados

Foram incluídos apenas estudos que atendessem aos seguintes critérios: (1) ensaios clínicos randomizados que investigassem o impacto das ADSCs; (2) estudos que utilizassem autoenxertos ou aloenxertos e fossem considerados elegíveis para análise; (3) pesquisas envolvendo pacientes com úlceras crônicas de extremidades de difícil cicatrização; e (4) avaliação de pelo menos um dos seguintes desfechos: taxa e tempo de cicatrização, taxa de amputação, mortalidade, infecções, qualidade de vida e dor. Foram excluídos estudos não randomizados, pesquisas em modelos animais, revisões, cartas ao editor ou artigos de opinião sem dados originais, ensaios que incluíssem pacientes tratados com células derivadas do tecido adiposo para feridas agudas ou feridas não localizadas em extremidades, bem como pacientes com doenças terminais ou contraindicações à terapia regenerativa. Não foram aplicadas restrições quanto ao tamanho da população estudada.

Os dados foram extraídos com base em critérios de busca e métodos de avaliação de qualidade predefinidos, com revisão em dois momentos distintos para minimizar o risco de exclusão indevida de estudos. A extração e a verificação dos dados foram realizadas de forma independente por dois autores, e eventuais divergências foram resolvidas por consenso entre três autores. Foram coletadas informações referentes às características dos pacientes, características das úlceras, tipo de intervenção realizada, taxa e tempo de cicatrização, além de resultados relacionados ao procedimento. Posteriormente, esses dados foram submetidos a revisão adicional e análise agrupada. Os desfechos primários avaliados foram a taxa e o tempo de cicatrização, enquanto os desfechos secundários incluíram o controle da dor e o perfil de segurança associado à terapia com ADSCs.

### Estratégia de busca

Realizou-se uma busca sistemática nas bases de dados PubMed, EMBASE, Cochrane Central Register of Controlled Trials e Scopus, desde o início de cada base de dados até dezembro de 2024, limitada a estudos publicados em língua inglesa. Foram utilizados os seguintes termos de busca: (*"Complex wounds"* OR *"Chronic wounds"* OR *"Non-healing wounds"* OR *"Pressure ulcers"* OR *"Diabetic foot ulcers"* OR *chronic* OR *ulcer* OR *diabetic*) AND (*"Adipose tissue therapy"* OR *"Adipose-derived stem cells"* OR *adipose* OR *"Fat grafting"* OR *"Cell therapy"* OR *"Stem cell therapy"* OR *"Regenerative therapy"* OR *"Regenerative medicine"* OR *"lipoaspirate cells"*) AND (*"Amputation"* OR *"Mortality"* OR *"Infection"* OR *healing* OR *"Tissue repair"* OR *"Tissue regeneration"* OR *"Wound closure"* OR *"Quality of life"*). As listas de referências dos estudos incluídos, bem como de revisões sistemáticas e metanálises relevantes, foram examinadas manualmente para identificar estudos adicionais potencialmente elegíveis que não tenham sido obtidos pela estratégia de busca inicial.

### Avaliação da qualidade

O risco de viés e a qualidade metodológica dos estudos incluídos foram avaliados por meio da ferramenta RoB 2 da Cochrane Collaboration para ensaios clínicos randomizados^13^. Cada estudo foi classificado como de baixo risco de viés, com algumas preocupações ou alto risco em cinco domínios específicos: processo de randomização, desvios das intervenções pretendidas, dados de desfechos ausentes, mensuração de desfechos e seleção dos resultados relatados. Além disso, foram construídos gráficos de funil para avaliar a possível presença de viés de publicação, relacionando as estimativas dos estudos com suas respectivas medidas de precisão.

### Análise estatística

Para comparar os desfechos binários entre os grupos de tratamento, foram calculadas *odds ratios* (ORs) com seus respectivos intervalos de confiança de 95% (IC95%). A heterogeneidade entre os estudos foi avaliada por meio do teste Q de Cochran e da estatística I^2^ de Higgins, enquanto o viés de publicação foi investigado utilizando-se o teste de Egger e gráficos de funil. Considerou-se baixa heterogeneidade quando p > 0,10 e I^2^ < 25%. As estimativas agrupadas foram calculadas utilizando o modelo de efeitos aleatórios de DerSimonian e Laird, adotando-se p < 0,05 como nível de significância estatística. O modelo de efeitos aleatórios foi empregado mesmo na ausência de heterogeneidade estatística significativa, devido à heterogeneidade clínica entre os estudos incluídos. Essa abordagem fundamenta-se na premissa de que os efeitos verdadeiros podem variar entre os estudos e que, se fosse possível realizar estimativas de efeito, todos os estudos seguiriam uma distribuição normal^14^. As análises estatísticas foram realizadas no *software* Review Manager 5.4 (Nordic Cochrane Centre, The Cochrane Collaboration, Copenhague, Dinamarca)^15^.

### Avaliação da certeza da evidência

A certeza da evidência foi avaliada por meio da abordagem Grading of Recommendations Assessment, Development and Evaluation (GRADE)^16^. A avaliação considerou cinco domínios: limitações dos estudos (risco de viés), inconsistência dos resultados, evidência indireta, imprecisão e viés de publicação. A certeza da evidência foi classificada em quatro níveis: alta, moderada, baixa ou muito baixa. Os resultados da avaliação foram apresentados em uma tabela de sumário dos achados.

## RESULTADOS

Como mostra a Figura 1, a estratégia de busca nos bancos de dados, complementada pela busca manual das listas de referências de revisões e metanálises relevantes, resultou na identificação inicial de 1.474 estudos. Após a remoção de duplicatas e a exclusão de registros irrelevantes com base na análise de títulos e resumos, 30 artigos foram selecionados para leitura na íntegra e avaliação segundo os critérios de inclusão e exclusão. Desses, 19 estudos foram excluídos pelos seguintes motivos: oito por não serem randomizados, cinco por consistirem em resumos de anais de estudos já incluídos ou por não atenderem aos critérios de elegibilidade, cinco por apresentarem desenhos de estudo sem resultados publicados e um por duplicidade. Ao final, 11 estudos foram incluídos na análise, totalizando 474 pacientes. Desses, 247 (52,1%) receberam tratamento com ADSC para úlceras crônicas, enquanto os 227 pacientes restantes (47,9%) foram submetidos ao tratamento convencional, com desbridamento, limpeza de ferida e curativos^17-27^.

**Figura 1:** Fluxograma Preferred Reporting Items for Systematic Reviews and Meta-Analyses (PRISMA) de triagem e seleção dos estudos.

A avaliação da qualidade metodológica por meio da ferramenta RoB 2 indicou que quatro ensaios clínicos apresentaram alto risco de viés, conforme ilustrado na Figura 2. No domínio referente ao processo de randomização, o estudo de Zollino et al.^17^ apresentou algumas preocupações, enquanto, Alinda et al.^26^ foi classificado como de alto risco, ambos em decorrência da falta de informações sobre a ocultação da sequência de alocação. No caso de Zollino et al.^17^, observou-se ainda alto risco de viés na mensuração dos desfechos, em razão da não descrição do cegamento dos avaliadores, o que limita a avaliação da adequação da randomização. Já o estudo de Namgoong et al.^18^ apresenta alto risco de viés na mensuração dos desfechos devido à avaliação não cega e ao uso de julgamentos clínicos subjetivos. Soma-se a isso a falha na ocultação da alocação (Domínio 1), que compromete a randomização, e a ausência de cegamento de participantes e aplicadores (Domínio 2), que gera preocupações adicionais, embora a análise por intenção de tratar tenha sido adequada. A falta de pré-registro do protocolo e do plano de análise também levanta dúvidas quanto à transparência e seleção de desfechos (Domínios 4 e 5). Em conjunto, esses fatores comprometem substancialmente a confiança nas estimativas de efeito do estudo. Por sua vez, a investigação conduzida por Uzun et al.^22^ apresentou alto risco de viés na seleção dos resultados relatados, ao apresentar apenas resultados estatisticamente significativos entre múltiplas subescalas sem correção para comparações múltiplas, além de alto risco de viés relacionado ao processo de randomização, devido às incertezas quanto à ocultação da alocação. Essas limitações metodológicas comprometem a validade interna dos respectivos estudos. No entanto, seu impacto sobre a avaliação global foi atenuado pela predominância de ensaios clínicos classificados como de baixo risco de viés.

**Figura 2:** Avaliação crítica dos estudos individuais de acordo com a ferramenta da Colaboração Cochrane para avaliação do risco de viés em estudos clínicos randomizados.

As características dos pacientes estão detalhadas na Tabela 1. Os estudos demonstraram heterogeneidade quanto à via de administração das ADSCs: dois ensaios clínicos utilizaram a aplicação tópica^23,26^, enquanto nove empregaram injeções locais^17-22,24,25,27^. Além disso, a origem das células variou entre os estudos: oito utilizaram tecido adiposo autólogo^17-22,24,25,27^, dois utilizaram tecido alogênico^22,23^, e um não especificou a fonte celular^26^. As regiões mais frequentemente utilizadas para a coleta do tecido adiposo foram o abdome inferior e lateral, bem como as partes interna e externa da coxa. O tempo de acompanhamento dos pacientes variou de 2 a 50 meses. Em relação à etiologia das úlceras, aproximadamente 71% dos casos estavam associados ao DM. As demais causas incluíram hanseníase, esclerose sistêmica, úlceras venosas, úlceras tróficas e úlceras arteriais, entre outras.

**Tabela 1:** Características dos estudos incluídos.

**Estudo**	**Pacientes**	**Idade (anos)**		**Sexo (M/F)**		**Etiologia das úlceras**			**Tamanho da ferida (cm^2^)**		**Duração da úlcera (semanas)**	**Tempo de acompanhamento (semanas)**	**ITB**
		ADSC	Controle	ADSC	Controle	ADSC	Controle		ADSC	Controle			
**Han et al. (2010)** **^27^**	52	66,5±7,5	68,4±8,7	15/11	14/12	DM			4,3±2,1	4,0±2,1	12,5±5,5	8	> 0,5
**Zollino et al. (2018)** **^17^**	16	74±6,7	68±12,8	5/3	5/3	75% UV/25% mista	62,5% UV/37,5% mista		31,3±59,0	29,0±42,0	≥ 36	24	> 0,85: UV; < 0,85: mista
**Del Papa et al. (2019)** **^24^** *	38	42 (21-69)^†^	37 (23-70) ^†^	2/23	0/13	Esclerose sistêmica			0,75 (0,65-0,82) ^†^	0,73 (0,62-0,84)†	ADSC: 11,4 (9,7-13,1) Controle: 8,0 (7,1-8,8)	8	N/A
**MiFrAADiF (2019)** **^19^**	114	69,0±11,6	71,6±10,8	45/12	41/16	DM			N/A	N/A	N/A	24	≥ 0,7
**Moon et al. (2019)** **^23^**	59	59,9±13,3	68,4±9,9	14/8	13/4	DM			2,0±0,9	2,8±2,0	35,6±75,2	12	0,7 < 1,3
**Smith et al. (2020)** **^21^** *	12	60,2 (45-78)	55,2 (41-69)	6/0	4/2	DM			3,4 (1,1-4,9)	5 (1,2-17,5)	49 (8-132)	12	≥0,3
**Tanios et al. (2021)** **^20^**	100	48,12±14,58	48,04±10,56	29/21	24/26	70% DM, 24% UT, 2% UV, 4% pós-trauma		84% DM, 16% UT	6,28±0,98	5,63±0,75	47,64±33,21	24	N/A
**Uzun et al. (2020)** **^22^**	20	57,5±8,4	57,2±4,5	6/4	6/4	DM			23,5±5,6	25,8±5,4	7,4±2,7	208,6‡	55% (< 0,8); 45% (≥ 0,8)
**Namgoong et al. (2022)** **^18^**	20	59,3±9,92	56,9±11,39	8/2	9/1	DM			5,66±4,74	4,66±6,86	≥ 6	16	N/A
**Alinda et al. (2023)** **^26^**	32	45,75±4,97	45,19±7,22	10/6	9/7	Hanseníase			1,67±1,52		> 6	8	N/A
**Thamm et al. (2023)** **^25^**	31	62±11	60±14	13/4	13/1	35,3% UV, 11,7% UA, 17,6% DM, 0.0% UPP, 47.0% outras	14,2% UV, 21,4% UA, 14,2% DM, 14,2% UPP, 42,8% outras		6,3±6,2	4,8±4	105±130	8,57	N/A

DM = diabetes mellitus; ITB = índice tornozelo-braquial; M/F = masculino/feminino; N/A = não se aplica; UA = úlceras arteriais; UPP = úlcera por pressão; UT = úlcera trófica; UV = úlcera venosa. Os valores são apresentados como média e desvio padrão.

* Os valores de idade, tamanho da ferida e duração da úlcera são apresentados como mediana e intervalo interquartil.

† Tamanho da ferida apresentado como mediana (intervalo interquartil), em centímetros.

‡ Tempo médio de seguimento do estudo: 208,6 semanas (intervalo: 113,0-217,3 semanas).

### Contribuição da ADSC na taxa de cicatrização de feridas

Dez estudos avaliaram a eficácia de terapia com ADSCs na taxa de cicatrização de feridas em comparação com o tratamento convencional, com períodos de acompanhamento variando de 6 a 24 meses. A cicatrização completa foi observada em 84,2% dos pacientes do grupo tratado com ADSCs, em comparação com 49,0% dos pacientes do grupo controle.

Na análise estratificada por subgrupos (úlceras associadas ao DM e úlceras cutâneas de etiologias diversas) observou-se que, no primeiro grupo, composto por seis ensaios clínicos, o efeito foi consistente e altamente significativo em favor da intervenção (OR 4,56; IC95% 2,40-8,67; p < 0,00001), sem evidência de heterogeneidade entre os estudos (I^2^ = 0%). No segundo subgrupo, o tratamento com ADSCs também foi associado a uma probabilidade significativamente maior de cicatrização em comparação ao grupo controle (OR 9,04; IC95% 2,60-31,36; p = 0,0005), embora tenha sido observada heterogeneidade moderada a alta entre os estudos (I^2^ = 52%).

A análise combinada dos ensaios clínicos confirmou o benefício global da terapia com ADSCs na cicatrização das úlceras (OR 5,77; IC95% 3,34-9,96; p < 0,00001), com baixa heterogeneidade entre os estudos (I^2^ = 9%). O teste de diferença entre subgrupos não revelou diferença estatisticamente significativa (p = 0,34), indicando que o efeito terapêutico foi consistente tanto em úlceras de etiologias diversas quanto naquelas especificamente associadas ao DM. Em conjunto, esses achados sugerem que a terapia com ADSCs representa uma estratégia promissora para a cicatrização de úlceras cutâneas, com evidência particularmente robusta e homogênea no contexto das úlceras diabéticas, conforme ilustrado na Figura 3. O teste de viés de Egger não demonstrou evidência de viés de publicação, apresentado coeficiente de viés de 0,593 (IC95% -1,363 a 2,550; p = 0,504). Esse resultado foi corroborado pela inspeção visual do gráfico de funil apresentado na Figura 4.

**Figura 3:** Gráfico de floresta demonstrando taxa de cicatrização significativamente maior das úlceras no grupo tratado com células-tronco derivadas do tecido adiposo. df = graus de liberdade (*degrees of freedom*); DM = diabetes melito; IC = intervalo de confiança; M-H = Mantel-Haenszel.

**Figura 4:** Gráfico de funil para avaliação de viés de publicação na taxa de cicatrização. DM = diabetes melito.

Ao estratificar a análise de pacientes com DM de acordo com a origem das células utilizadas nos ensaios clínicos, observou-se que o subgrupo tratado com ADSCs autólogas (quatro estudos; 190 participantes) apresentou efeito significativo em favor da intervenção (OR 5,69; IC95% 2,72-11,90; p < 0,00001; I^2^ = 0%). No subgrupo tratado com células alogênicas (dois estudos; 59 participantes), observou-se tendência favorável ao tratamento, com significância estatística limítrofe (OR 3,49; IC95% 0,99-12,29; p = 0,05; I^2^ = 0%). O teste de diferença entre subgrupos não evidenciou diferença estatisticamente significativa (p = 0,51; I^2^ = 0%), sugerindo que o efeito terapêutico das ADSCs não diferiu significativamente entre as fontes celulares avaliadas.

Após a exclusão dos ensaios clínicos classificados como de alto risco pela ferramenta RoB 2, a análise de sensibilidade demonstrou que a significância estatística e a estimativa do efeito permaneceram inalteradas, com OR combinado de 7,79 (IC95% 3,25-18,67; p < 0,00001; Z = 4,60). A heterogeneidade entre os estudos permaneceu baixa (I^2^ = 23,3%; p = 0,25), indicando consistência dos resultados. Na análise estratificada por etiologia, o subgrupo de úlceras cutâneas de etiologias múltiplas (dois estudos; 138 participantes por grupo) apresentou OR de 26,11 (IC95% 1,59-429,31), sugerindo benefício da intervenção. Já no subgrupo de úlceras diabéticas (quatro estudos; 208 participantes), o efeito estimado foi de OR = 4,86 (IC95% 2,43-9,74; p < 0,0001), sem evidência de heterogeneidade entre os estudos (I^2^ = 0%; p = 0,55), reforçando a robustez da evidência nessa população.

A análise individual das ORs demonstrou que cinco estudos apresentaram resultados estatisticamente significativos em favor da terapia com ADSCs^19,20,24,26,27^. Destaca-se o estudo de Del Papa et al.^24^, que relatou OR de 138,00 (IC95% 11,33-1.680,96), indicando um efeito substancial da intervenção, embora com um intervalo de confiança amplo. De forma semelhante, Han et al.^27^ reportaram OR de 33,73 (IC95% 1,85-614,71), reforçando os benefícios da intervenção. Além disso, os estudos MiFrAADiFL^19^ e de Tanios et al.^20^ apresentaram ORs de 4,70 (IC95% 1,98-11,44) e 7,67 (IC95% 2,38-24,65), respectivamente, demonstrando benefício estatisticamente significativo da terapia com ADSCs. Por outro lado, Moon et al.^23^ (OR 4,00; IC95% 0,95-16,92) e Smith et al.^21^ (OR 2,50; IC95% 0,16-38,60) apresentaram estimativas de baixa precisão, evidenciadas pelos amplos intervalos de confiança, possivelmente devido ao tamanho reduzido das amostras.

### Impacto da ADSC no tempo de cicatrização

Sete estudos forneceram dados suficientes para a análise do tempo de cicatrização. No grupo tratado com ADSCs, composto por 183 indivíduos, o tempo médio de cicatrização variou de 31 a 122 dias, enquanto no grupo controle, com 178 participantes, variou de 39 a 171 dias. A diferença média ponderada combinada indicou redução média de 19,39 dias no tempo de cicatrização em favor do grupo de intervenção (IC95% -30,17 a -8,61; p = 0,0004), conforme apresentado na Figura 5.

**Figura 5:** Gráfico de floresta demonstrando tempo médio de cicatrização das úlceras significativamente menor no grupo tratado com células-tronco derivadas do tecido adiposo. df = graus de liberdade (*degrees of freedom*); DP = desvio padrão; IC = intervalo de confiança; IV = variância inversa (*inverse variation*) ; M-H = Mantel-Haenszel.

Observou-se heterogeneidade significativa na análise do tempo de cicatrização, com Tau^2^ = 169,96. A estatística de Cochran Q foi de 77,64 [graus de liberdade (*degrees of freedom* (df) = 6; p < 0,00001], e o índice I^2^ foi de 92% (IC95% 81,4%-98,4%), confirmando elevada heterogeneidade estatística e considerável inconsistência entre os estudos. O gráfico de funil (Figura 6) evidenciou ampla dispersão das estimativas de efeito, compatível com a elevada heterogeneidade observada.

**Figura 6:** Gráfico de funil para avaliação de viés de publicação no tempo médio de cicatrização.

Na análise estratificada por subgrupos, considerando exclusivamente as úlceras diabéticas (cinco estudos; 236 participantes), observou-se diferença média de -12,44 dias no tempo de cicatrização em favor da terapia com ADSC (IC95% -18,33 a -6,55; p < 0,0001), embora com heterogeneidade substancial entre os estudos (I^2^ = 68%; p = 0,01). Na estratificação por origem celular, o subgrupo tratado com ADSCs autólogas (quatro estudos; 197 participantes) apresentou diferença média de -13,52 dias (IC95% -23,39 a -3,64; p = 0,007), também com heterogeneidade substancial (I^2^ = 76%; p = 0,006). Na análise de sensibilidade, após a exclusão dos estudos classificados como de alto risco de viés, o efeito permaneceu estatisticamente significativo, com diferença média de -15,55 dias (IC95% -30,11 a -0,98; p = 0,04), embora a heterogeneidade tenha permanecesse elevada (I^2^ = 96%; p < 0,00001). Em conjunto, esses achados evidenciam expressiva variabilidade entre os estudos, possivelmente relacionada a diferenças nos protocolos de aplicação das células e na duração do acompanhamento dos pacientes.

Individualmente, o estudo de Zollino et al.^17^ apresentou a maior diferença média no tempo de cicatrização (-49,00 dias; IC95% -90,45 a -7,55), embora com um intervalo de confiança amplo. Os estudos de Tanios et al.^20^ (-42,00 dias; IC95% -49,34 a -34,66) e Namgoong et al.^18^ (-21,70 dias; IC95% -31,94 a -11,46) também demonstraram reduções estatisticamente significativas no tempo de cicatrização. Em contrapartida, o estudo MiFrAADiF^19^ não identificou diferença significativa entre os grupos (diferença média = 0,00 dias; IC95% -13,24 a 13,24).

### Contribuição da ADSC na modulação da dor

Cinco trabalhos avaliaram os efeitos da terapia com ADSCs sobre a dor, utilizando a Escala Visual Analógica (EVA), a Escala Numérica de Avaliação da Dor (*Numeric Rating Scale*, NRS) ou o domínio de dor do questionário *Short Form Health Survey* (SF-36)^17,19,22,24,25^. Desses, dois estudos demonstraram resultados estatisticamente significativos em favor da terapia com ADSCs^17,24^.

Del Papa et al.^24^ observaram redução superior a 50% da intensidade de dor em 21 dos 25 pacientes tratados com extrato de tecido adiposo, enquanto nenhum dos 13 pacientes do grupo controle apresentou melhora comparável após 8 semanas de acompanhamento (p < 0,00001; teste exato de Fisher). No estudo de Zollino et al.^17^, a pontuação da NRS diminuiu para 2,7±2,0 na primeira semana após a aplicação de células autólogas do tecido adiposo, em comparação com 6,6±3,0 no grupo controle (p < 0,01).

Por outro lado, o estudo MiFrAADiF^19^ não encontrou diferenças significativas entre os grupos em nenhuma das avaliações de acompanhamento. Observou-se uma redução da dor ao longo do tempo (p < 0,001); no entanto, o tratamento com tecido adiposo microfragmentado também contribuiu significativamente para esse desfecho (p < 0,05). De modo semelhante, Thamm et al.^25^ e Uzun et al.^22^ relataram discreta redução da dor em ambos os grupos durante o seguimento, sem que as diferenças observadas alcançassem significância estatística.

### Perfil de segurança relacionado a terapia com ADSCs

Dos ensaios clínicos selecionados, nove forneceram dados suficientes para a avaliação de eventos adversos relacionados ao procedimento. Dois estudos relataram eventos adversos no grupo tratado com ADSCs^17,19^, enquanto os demais não identificaram complicações associadas à intervenção^18,20,22-24,26,27^. No total, foram registrados três eventos adversos entre os 222 pacientes tratados com ADSCs, em comparação com nenhum evento entre os 200 pacientes do grupo controle.

Em um dos casos, foi observada dermatite perilesional logo após a administração das células, com resolução espontânea na segunda semana de acompanhamento^17^. No estudo MiFrAADiF, foram relatados dois hematomas da parede abdominal no local da coleta do tecido adiposo: um foi tratado com curativo compressivo, enquanto o outro exigiu incisão cirúrgica para hemostasia do tecido subcutâneo. Ambos os pacientes faziam uso de anticoagulantes orais.

Em um estudo de três braços conduzido por Smith et al.^21^, um paciente desenvolveu uma coleção superficial de fluido ao longo do trajeto da cânula utilizada para o enxerto, necessitando de drenagem. No entanto, os autores não especificaram em qual braço do estudo o paciente estava alocado, se no grupo tratado com tecido adiposo, (intervenção de interesse desta revisão), ou no terceiro braço, que recebeu enxerto de gordura associado a plasma rico em plaquetas.

A análise mostrou uma OR de 4,07 eventos (IC95% 0,42-38,91), sugerindo numericamente maior ocorrência de complicações no grupo que utilizou ADSCs; entretanto, a ampla amplitude do intervalo de confiança indica considerável imprecisão da estimativa. O teste de efeito geral (Z = 1,23; p = 0,22) não demonstrou diferença estatisticamente significativa entre os grupos. Além disso, não foi observada heterogeneidade significativa na análise da segurança entre os estudos (Q = 0,02; df = 1; p = 0,89; I^2^ = 0%), conforme ilustrado na Figura 7.

**Figura 7:** Gráfico de floresta demonstrando que a terapia com células-tronco derivadas do tecido adiposo demonstrou perfil de segurança favorável, sem aumento significativo do risco de complicações em comparação com a terapia padrão. df = graus de liberdade (*degrees of freedom*); IC = intervalo de confiança; M-H = Mantel-Haenszel.

Portanto, embora eventos adversos tenham sido relatados no grupo tratado com ADSCs, as evidências disponíveis não demonstram um aumento significativo do risco de complicações em comparação com o tratamento padrão.

### Avaliação da certeza da evidência

A certeza da evidência foi avaliada por meio da abordagem GRADE para os desfechos taxa de cicatrização, tempo de cicatrização e segurança (Tabela 2). Para a taxa de cicatrização, a análise principal apresentou certeza moderada, rebaixada por evidência indireta decorrente da heterogeneidade clínica relacionada à etiologia das úlceras e aos protocolos de intervenção. Na análise restrita a pacientes com DM, a certeza permaneceu moderada, rebaixada por imprecisão devido ao número reduzido de eventos (< 300). Quanto ao tempo de cicatrização, tanto a análise envolvendo úlceras de diferentes etiologias quanto a análise específica para DM apresentaram certeza muito baixa, em razão da heterogeneidade elevada (I^2^ > 50%), não explicada pela análise de subgrupos. Para o desfecho segurança, a certeza da evidência também foi classificada como muito baixa, devido a inconsistência muito grave (sobreposição dos intervalos de confiança), evidência indireta e imprecisão muito grave (diamante da estimativa combinada cruzando a linha de nulidade e apresentando intervalo de confiança muito amplo).

**Tabela 2:** Avaliação da certeza da evidência segundo a abordagem Grading of Recommendations Assessment, Development and Evaluation (GRADE).

**Avaliação da certeza**							**Nº de pacientes**		**Efeito**		**Certeza**	**Importância**
**Nº dos estudos**	**Delineamento do estudo**	**Risco de viés**	**Inconsistência**	**Evidência indireta**	**Imprecisão**	**Outras considerações**	**ADSC**	**Controle**	**Relativo (IC95%)**	**Absoluto (IC95%)**		
**Taxa de cicatrização de úlceras de extremidades sem discriminação por etiologia**												
10	Ensaios clínicos randomizados	Não grave	Não grave	Grave^*^	Não grave	Nenhum	192/228 (84,2%)	99/202 (49,0%)	OR 5,77 (3,34-9,96)	357 eventos a mais por 1.000 (de 272 a 415 a mais)	⊕⊕⊕◯ Moderada*	Crítico
**Taxa de cicatrização de úlceras em pacientes com DM**												
6	Ensaios clínicos randomizados	Não grave	Não grave	Não grave	Grave^†^	Nenhum	107/129 (82,9%)	60/115 (52,2%)	OR 4,56 (2,40-8,67)	311 eventos a mais por 1.000 (de 202 a 383 a mais)	⊕⊕⊕◯ Moderada^†‡^	Crítico
**Tempo de cicatrização de úlceras de extremidades sem discriminação por etiologia**												
7	Ensaios clínicos randomizados	Não grave	Muito grave^‡^	Grave^*^	Não grave	Nenhum	181	171	-	MD: 19,39 dias a menos (30,17 a menos a 8,61 a menos)	⊕〇〇〇 Muito baixa^*‡^	Importante
**Tempo de cicatrização de úlceras em pacientes com DM**												
5	Ensaios clínicos randomizados	Não grave	Muito grave^‡^	Não grave	Grave^†^	Nenhum	123	113	-	MD: 12,44 dias a menos (18,33 a menos a 6,55 dias a menos)	⊕〇〇〇 Muito baixa†‡	Importante
**Complicações relacionada ao uso de ADSC**												
9	Ensaios clínicos randomizados	Não grave	Muito grave§	Grave^*^	Muito grave	Nenhum	3/63 (4,8%)	0/58 (0,0%)	OR 4,07 (0,42-38,91)	0 por 1.000 pacientes (0 a 0)	⊕〇〇〇 Muito baixa*§||	Importante

IC = intervalo de confiança; MD = diferença média (*mean difference*); OR = *odds ratio*.

* População composta por pacientes com úlceras de diferentes etiologias (diabéticas, venosas, arteriais, autoimunes e hansênicas) e submetidos a diferentes métodos de administração de ADSC (aplicação tópica *versus* injeção local; autólogo *versus* alogênico).

† Menos de 300 eventos.

‡ Heterogeneidade elevada (I^2^ > 50%).

§ Ampla sobreposição dos intervalos de confiança entre os estudos.

|| O diamante da estimativa combinada cruza a linha de nulidade e apresenta amplo intervalo de confiança.

## DISCUSSÃO

As células-tronco mesenquimais (MSCs) emergiram como uma estratégia terapêutica promissora para o tratamento de feridas crônicas^28,29^. Essas células podem ser obtidas de diferentes fontes, incluindo medula óssea, tecido adiposo e cordão umbilical. As ADSCs constituem uma população de MSCs isoladas do tecido adiposo. Diversos estudos, tanto *in vitro* quanto *in vivo*, demonstraram os benefícios terapêuticos das MSCs, tais como a liberação de citocinas pró-angiogênicas, o estímulo à epitelização e à formação de tecido de granulação, além de efeitos anti-inflamatórios e antiapoptóticos que favorecem o processo de cicatrização de úlceras^19,30^.

As ADSCs e terapias relacionadas, como folhas celulares, enxertos autólogos, enxertos alogênicos e exossomos, podem melhorar significativamente a cicatrização de feridas ao promover a regeneração tecidual, a neovascularização e a formação de tecido de granulação, além de reduzir a inflamação e melhorar a qualidade da pele em úlceras diabéticas, venosas, tróficas e por pressão^30-33^.

Esta metanálise demonstrou que 83,3% dos pacientes tratados com ADSCs alcançaram cicatrização completa, em comparação com 48,5% daqueles submetidos ao tratamento convencional baseado em desbridamento, limpeza da ferida e curativos, evidenciando diferença estatisticamente significativa. Achados semelhantes foram relatados por Elsharkawi et al.^5^ em uma metanálise que avaliou a cicatrização de úlceras em pacientes com DM. Nesse estudo, conduzido com 189 pacientes acompanhados por até 12 meses, a cicatrização completa foi observada em 83,5% dos indivíduos tratados com ADSCs, em comparação com 52% dos participantes do grupo controle. A tendência favorável à intervenção também foi observada em úlceras de outras etiologias. Del Papa et al.^24^, ao avaliarem pacientes com esclerose sistêmica, verificaram que 92% dos indivíduos tratados com enxerto de tecido adiposo apresentaram cicatrização das úlceras digitais após 8 semanas, em contraste com apenas 7,7% no grupo controle.

A terapia com ADSC tem demonstrado resultados promissores no tratamento de feridas em pacientes com DM^34^. Han et al.^27^ relataram cicatrização completa em todos os pacientes tratados com células derivadas de lipoaspirado humano processado, em comparação com 62% dos participantes do grupo controle. Moon et al.^23^, por sua vez, utilizaram ADSCs alogênicas derivadas de doadores saudáveis incorporadas em folhas de hidrogel para o tratamento de úlceras do pé diabético, observando fechamento completo da lesão em 82% dos pacientes tratados, em comparação com 53% no grupo controle após 12 semanas de acompanhamento (p = 0,053). Em conjunto, esses achados sugerem que as ADSCs, particularmente quando administradas na forma de folhas celulares, podem favorecer a cicatrização por meio da promoção da vascularização, da redução da fibrose e da restauração da arquitetura cutânea^35^.

Embora a análise estatística tenha demonstrado baixa heterogeneidade para a taxa de cicatrização (I^2^ = 9%), é importante considerar a heterogeneidade clínica entre os estudos, especialmente em relação à etiologia das úlceras. Os mecanismos fisiopatológicos envolvidos na cicatrização diferem substancialmente entre úlceras venosas, arteriais, diabéticas, hansênicas e autoimunes, refletindo processos biológicos distintos. Dessa forma, o agrupamento dessas etiologias em uma única análise, ainda que estatisticamente homogênea, pode mascarar diferenças clinicamente relevantes na resposta terapêutica. O benefício observado pode não ser uniforme entre os diferentes subgrupos, sendo plausível que a magnitude do efeito varie de acordo com a etiologia da úlcera. Assim, a extrapolação dos resultados para todos os tipos de feridas deve ser realizada com cautela, especialmente considerando as limitações inerentes às análises estratificadas, que envolvem tamanhos amostrais reduzidos.

Adicionalmente, a magnitude do efeito observada (OR = 5,77) deve ser interpretada com cautela. Estudos com intervalos de confiança mais amplos e menor tamanho amostral tenderam a apresentar estimativas de efeito mais extremas, padrão compatível com a possível presença de efeitos de estudo pequenos^36^. Embora esse fenômeno possa estar relacionado a limitações metodológicas e à publicação preferencial de resultados positivos, os dados disponíveis não permitem confirmar essa hipótese. Dessa forma, não se pode descartar a possibilidade de que o benefício observado esteja parcialmente superestimado.

O grupo tratado com ADSC apresentou menor tempo de cicatrização, com diferença média padronizada (*standardized mean difference*, SMD) global de -19,35 (IC95% -30,12 a -8,58). O estudo de Tanios et al.^20^ destacou-se por apresentar a maior magnitude de efeito, com SMD de -42,03 (IC95% -49,37 a -34,69). Nesse estudo, o tempo médio de cicatrização foi de 7,87±2,50 semanas no grupo tratado com ADSCs, em comparação com 13,87±2,84 semanas no grupo controle (p = 0,000), sendo as injeções administradas a cada 3 semanas.

Intervenções como as descritas por Tanios et al.^20^ e Namgoong et al.^18^ demonstraram potencial para acelerar a cicatrização e proporcionar benefícios clinicamente relevantes. Na análise restrita a pacientes com úlceras diabéticas, a terapia com ADSCs apresentou benefício consistente, com redução aproximada de 12 dias no tempo de cicatrização. Esse achado possui importante relevância clínica, considerando que o pé diabético está entre as principais causas de morbidade, hospitalização e amputações não traumáticas^37^. A redução do tempo de cicatrização pode contribuir para a melhora da qualidade de vida dos pacientes e para a diminuição dos custos em saúde.

A alta heterogeneidade observada (I^2^ = 68%) na metanálise de pacientes com DM para o desfecho tempo de cicatrização sugere a presença de múltiplas fontes de variabilidade, incluindo diferenças na gravidade das úlceras, nos protocolos terapêuticos adjuvantes (desbridamento, antibioticoterapia e descarga de pressão), no tamanho amostral e na duração do seguimento. Estudos que relataram menor tempo de cicatrização no grupo controle podem ter incluído pacientes com úlceras menos graves, enquanto aqueles com pior prognóstico basal parecem ter obtido maior benefício absoluto com a intervenção. O estudo MiFrAADiF^19^, único a não demonstrar benefício da terapia, pode refletir particularidades populacionais ou metodológicas; entretanto, sua inclusão não modificou a direção do efeito global, contribuindo principalmente para o aumento da variabilidade entre os estudos.

Apesar da variabilidade observada, a consistência direcional dos achados – com todos os estudos, exceto um, favorecendo a intervenção – reforça a evidência de benefício da terapia com ADSCs na redução do tempo de cicatrização de úlceras diabéticas. O efeito mostrou-se mais pronunciado nas análises envolvendo células autólogas; contudo, a elevada heterogeneidade observada tanto na análise global quanto nas análises por subgrupos reduz a precisão da estimativa combinada. Além disso, o número limitado de estudos impossibilitou a realização de análises de meta-regressão capazes de investigar fontes específicas de inconsistência, como tamanho da úlcera, presença de infecção e controle glicêmico.

Um desfecho secundário relevante observado por Zollino et al.^17^ e Del Papa et al.^24^ foi a redução significativa da dor, evidenciada por melhora imediata nas escalas NRS e EVA, respectivamente, após a terapia celular. A úlcera venosa crônica está associada a importante comprometimento da qualidade de vida, em grande parte devido à dor persistente e de difícil controle. Nesse contexto, a terapia com células-tronco pode modular a cascata inflamatória local por meio da secreção de fatores bioativos, exercendo efeitos anti-inflamatórios e imunomoduladores. Esse mecanismo é compatível com a hipótese do secretoma e com a capacidade das células-tronco de modificar o microambiente da ferida em favor da reparação tecidual^31^.

Por outro lado, o estudo MiFrAADiF^19^ não identificou diferenças significativas entre os grupos quanto à redução da dor, observando que essa melhora foi predominantemente associada ao tempo (p < 0,001). No entanto, os pacientes do grupo de tratamento apresentaram redução da dor mais precocemente durante o tratamento. Segundo os autores, a ausência de diferenças entre os grupos pode estar relacionada à elevada prevalência de polineuropatia distal em pacientes com DM, o que dificulta a distinção entre dor neuropática e dor relacionada à ferida^19,38^. Estudos em pacientes com osteoartrite demonstraram que a injeção intra-articular de tecido adiposo microfragmentado está associada a melhora significativa dos escores na escala VAS^39^.

Em relação à segurança, as diferentes modalidades de administração de ADSCs (incluindo injeções, folhas celulares e aplicação tópica), apresentaram perfil de segurança favorável. No estudo MiFrAADiF^19^, além dos hematomas da parede abdominal previamente descritos, foram registradas sete mortes durante o período de acompanhamento; contudo, nenhuma foi considerada relacionada ao tratamento. Alguns ensaios clínicos também relataram redução da incidência de infecções nas úlceras tratadas. Tanios et al.^20^ observaram infecção em apenas 6% dos pacientes do grupo tratado, em comparação com 28% dos participantes do grupo controle (p = 0,000). Esse efeito pode estar relacionado às propriedades biológicas das células-tronco e ao aumento da vascularização promovido pela angiogênese^19,30^.

No ensaio clínico relatado por Han et al.^27^, dois participantes foram excluídos devido a infecções clinicamente evidentes ocorridas 5 e 6 semanas após o tratamento. Moon et al.^23^ relataram eventos adversos graves, incluindo celulite em locais não relacionados à úlcera tratada, parestesia, descontrole glicêmico e parada cardíaca. Por sua vez, Uzun et al.^22^ observaram complicações em 15% dos voluntários, incluindo infecções recorrentes, necrose e amputações menores. Importante destacar que nenhum desses eventos foi considerado relacionado a terapia com ADSCs ou aos curativos empregados nos estudos.

A avaliação do custo-efetividade das ADSCs no tratamento de úlceras crônicas de extremidades deve considerar não apenas o custo inicial da intervenção, mas também o custo total do episódio de cuidado. Estudos de avaliação econômica sugerem que produtos celulares e teciduais podem ser custo-efetivos quando analisados em horizonte temporal apropriado. Carter e Fife^40^ estabeleceram parâmetros objetivos para essa avaliação, indicando que intervenções com custo ≤ US$ 140 por cm^2^ podem ser consideradas dominantes (isto é, menos custosas e associadas a melhores desfechos) em comparação ao cuidado padrão. Em contrapartida, valores > US$ 430 por cm^2^ excedem o limiar de custo-efetividade de US$ 100.000 por ano de vida ajustado pela qualidade (*quality-adjusted life years*, QALY), tornando sua adoção menos justificável do ponto de vista econômico. Adicionalmente, Nherera & Banerjee^41^ demonstraram, em um modelo com horizonte temporal de 1 ano, que todos os produtos celulares avaliados foram custo-efetivos em comparação ao cuidado padrão, com custos totais variando de US$ 10.907 a US$ 19.498, em contraste com US$ 19.862 para o tratamento convencional. Esses resultados sugerem que o investimento inicial pode ser compensado pela redução de complicações como infecções e amputações.

No contexto específico das ADSCs, os achados de Uzun et al.^22^ e Smith et al.^21^ são consistentes com essa perspectiva. Embora os custos imediatos da terapia sejam mais elevados, benefícios potenciais, como retorno mais precoce às atividades laborais, menor incidência de osteomielite e redução das taxas de amputação, podem resultar em economia global para o sistema de saúde. Entretanto, a variabilidade dos custos observada entre produtos similares reforça a necessidade de avaliações econômicas incorporadas aos ensaios clínicos, de modo a determinar se as ADSCs se enquadram nos limiares de custo-efetividade estabelecidos (US$ 140-US$ 430/cm^2^) e identificar as subpopulações de pacientes (como aqueles com úlceras extensas ou refratárias) nas quais o benefício clínico justifica o investimento.

À luz das evidências analisadas nesta revisão, e considerando as limitações identificadas, observou-se benefício terapêutico associado ao uso de ADSC no tratamento de úlceras crônicas de extremidades. A certeza da evidência, avaliada pela abordagem GRADE, foi moderada para o desfecho de cicatrização, sendo mais robusta entre pacientes com úlceras diabéticas tratados com células autólogas. Para a taxa de cicatrização, a evidência foi classificada como baixa, em razão das limitações inerentes a presente revisão e do número reduzido de ensaios clínicos randomizados disponíveis. Os estudos classificados como de maior risco de viés não influenciaram substancialmente os resultados da metanálise. Entre as principais limitações deste estudo destacam-se o reduzido tamanho amostral e o agrupamento de diferentes tipos de úlceras crônicas de extremidades sem caracterização etiológica detalhada, fatores que podem ter contribuído para heterogeneidade clínica residual.

## CONCLUSÃO

O uso de ADSCs no tratamento de úlceras crônicas de extremidades mostra-se promissor, com potencial para reduzir significativamente o tempo de cicatrização e aumentar a taxa de cicatrização das lesões, particularmente em úlceras diabéticas e em intervenções que utilizam células autólogas. As evidências atualmente disponíveis não indicam aumento significativo do risco associado aos procedimentos de coleta e administração das ADSCs. Entretanto, a heterogeneidade metodológica entre os estudos requer cautela na interpretação dos resultados e na extrapolação dos resultados para diferentes populações clínicas. Embora quatro ensaios clínicos terem sido classificados como de alto risco de viés pela ferramenta RoB 2, seus resultados foram consistentes com aqueles observados em estudos com baixo risco de viés, reforçando a direção favorável dos achados. Ainda assim, são necessários estudos adicionais, com amostras maiores, delineamento randomizado, maior rigor metodológico e protocolos padronizados, para confirmar esses resultados e definir com maior precisão o papel das ADSCs no manejo de feridas crônicas.

## Data Availability

All data generated or analyzed during this study are included in this published article and/or its supplementary material.
